# Enhancing DC cancer vaccine by allogeneic MHC class II expression and Treg depletion

**DOI:** 10.1172/jci.insight.189024

**Published:** 2025-07-15

**Authors:** Noriko Seishima, William Becker, Purevdorj B. Olkhanud, Hoyoung M. Maeng, Miguel A. Lopez-Lago, William V. Williams, Jay A. Berzofsky

**Affiliations:** 1 Vaccine Branch, Center for Cancer Research, National Cancer Institute, NIH, Bethesda, Maryland, USA.; 2 BriaCell Therapeutics Corp., Philadelphia, Pennsylvania, USA.

**Keywords:** Immunology, Oncology, Cancer immunotherapy

## Abstract

We assessed the therapeutic efficacy of a semiallogeneic dendritic cell (DC) vaccine in comparison to a syngeneic one for suppression of B16-F10 and TC-1 tumors. Syngeneic bone marrow–derived DCs (BMDCs) were generated from C57BL/6J mice and semiallogeneic BMDCs with a mutation in either MHC class I or II were generated from B6.C-*H2-K^bm1^*/ByJ or B6(C)-*H2-Ab1^bm12^*/KhEgJ mice, respectively. We demonstrated in vivo and in vitro that the MHC class II semiallogeneic BMDC vaccine had superior efficacy over the syngeneic and the MHC class I semiallogeneic BMDC vaccine, providing allogeneic CD4^+^ T cell help to enhance the antitumor CD8^+^ T cell response through allogeneic stimulation by the mutant MHC class II molecules. We discovered that this help was induced only at an early stage of tumor growth and at a later stage of tumor growth; combining our BMDC vaccine with Treg depletion enhanced tumor suppression. We demonstrated the improved efficacy of a semiallogeneic BMDC vaccine that kept tumor-peptide presentation intact on syngeneic MHC class I molecules so that mutant MHC class II could provide allogeneic help. This strategy should enable promising new DC-based cancer immunotherapies, offering an alternative to autologous DC vaccines by incorporating allogenicity as an adjuvant.

## Abstract 2

Peptide-pulsed MHC class II semi-allogeneic dendritic cell vaccine shows superior efficacy over syngeneic vaccine and is enhanced by late Treg depletion in mouse cancer models

## Graphical abstract

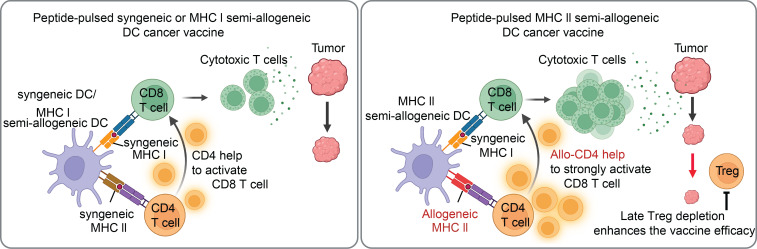

## Introduction

Dendritic cells (DCs) are the most potent antigen-presenting cells (APCs), uniquely capable of presenting exogenous antigens on MHC class I to activate CD8^+^ cytotoxic T lymphocytes (CTLs), in addition to presenting them on MHC class II to CD4^+^ T cells (1, 2). Since the 1990s, DC-based vaccines have been widely studied in preclinical and clinical settings, with various strategies developed to enhance their efficacy (3–6).

DC vaccination aims to elicit antigen-specific effector and memory T cells to promote tumor rejection (5). However, tumors suppress DC maturation in vivo, limiting antigen presentation (7–9). To overcome this, most approaches use autologous DCs matured ex vivo with tumor antigens, away from the immunosuppressive tumor microenvironment (10, 11). DC vaccine protocols vary in origin of cells, culture, maturation, and delivery methods (6). Although DCs in circulation represent only about 1% of PBMCs (11), it was recently described that it is possible to obtain high numbers of plasmacytoid DCs or myeloid DCs without an extensive culture (12–14). Nevertheless, the most common DC source is monocyte-derived autologous DCs from peripheral blood or bone marrow (BM), requiring 5–7 days of cytokine-based differentiation (15–17). These autologous DC-based vaccines as a monotherapy have demonstrated safety in cancer patients with some success, but there is a clear need to improve the response in both efficacy and durability (18). Combining DC vaccines with chemotherapy, radiotherapy, and other immune-modulating therapies such as immune checkpoint inhibitors (ICIs) demonstrated increased antitumor responses (18–22).

One strategy to boost immunogenicity is the use of semiallogeneic DC hybrids, created by fusing syngeneic and allogeneic cells to coexpress both MHC types (23–30). This is facilitated by the closer proximity of these allospecific Th cells and antigen-specific CTL brought about by common APCs (23, 29). Allogeneic MHC class II molecules may recruit high-frequency alloreactive CD4^+^ T cells, enhancing peptide-specific CTL responses through self-MHC presentation (25, 26, 31). However, these earlier fusion-based studies did not differentiate between alloresponses induced by MHC class I or class II molecules.

An additional advantage of semiallogeneic DC vaccines is that they do not require derivation from each patient, which allows for “off-the-shelf” DCs. The vaccine would instead be selected to be semiallogeneic to retain shared MHC antigens of the patient that can present tumor antigens to the patient’s T cells. Semiallogeneic DC vaccines, by amplifying CD4^+^ T cell help, could improve both quantity and quality of the CD8^+^ CTLs as well as the CD4^+^ Th cell function, thereby enhancing overall efficacy of DC-based immunotherapy as a monotherapy or in combination regimens.

In this study, we aimed to investigate whether a therapeutic MHC class I or II semiallogeneic DC vaccine was superior to a syngeneic DC vaccine in suppressing tumor growth. We used semiallogeneic DCs that had nonmutant H2Db MHC class I to maintain the binding and presentation of antigenic peptide by an MHC class I molecule syngeneic to the recipient of the DC to stimulate the recipient’s antigen-specific CTLs directly. This allowed us to evaluate the additional allogeneic help provided by either of the mutant MHC class I or II molecules separately.

Here, we report an efficacious peptide-pulsed semiallogeneic DC vaccine study in mouse cancer models and found that the MHC class II semiallogeneic DC vaccine was superior to the syngeneic WT or the MHC class I semiallogeneic DC vaccine. The peptide-pulsed MHC class II semiallogeneic DC vaccine induced alloantigen-specific CD4^+^ Th cells to improve the antigen-specific CD8^+^ CTL response. In exploring the mechanisms of this allogeneic CD4^+^ T cell help in tumor challenge studies, we discovered that this help was induced only at an early stage of tumor growth by the peptide-pulsed MHC class II semiallogeneic DC vaccine. At a later stage of tumor growth, combining our DC vaccine with Treg depletion directly or through CD4^+^ T cell depletion further enhanced tumor suppression. We believe that understanding the mechanisms underlying the MHC class II semiallogeneic DC vaccine, including identifying related stimulator or suppressor CD4^+^ T cells or altering the tumor microenvironment, will pave the way for promising new DC-based cancer immunotherapies beyond what could be accomplished by the cell fusion approach.

## Results

### Syngeneic, MHC class I semiallogeneic and MHC class II semiallogeneic BMDCs express MHC class I/II and costimulatory molecules similarly, but MHC class II semiallogeneic BMDCs can help WT CD4^+^ T cells proliferate.

**Figure 1:**
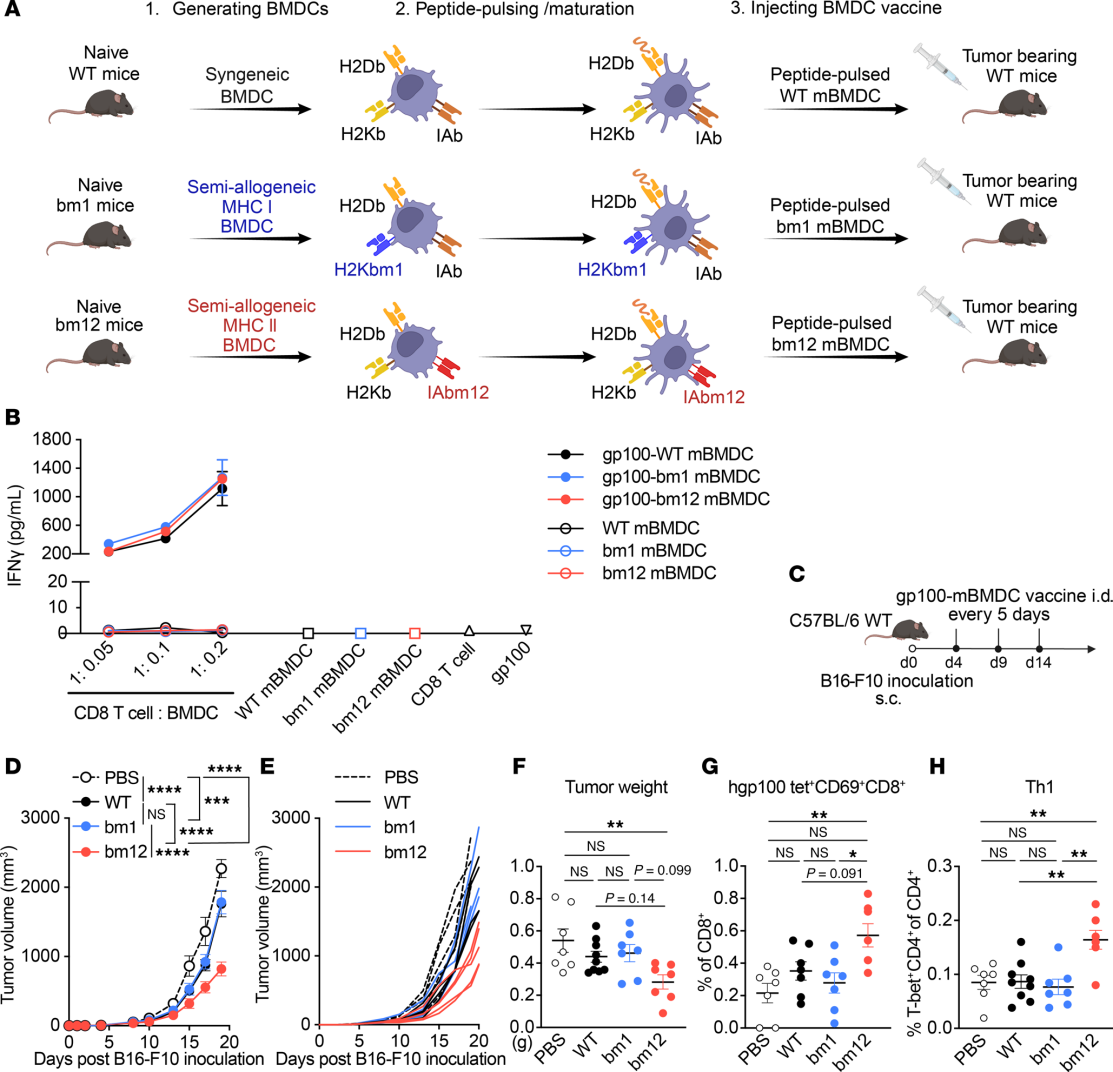
Syngeneic and semiallogeneic BMDCs present H2Db-restricted peptide to CD8^+^ T cells similarly, but the gp100_25–33_ peptide–pulsed MHC class II semiallogeneic BMDC vaccine is superior for delaying of B16-F10 tumor growth and improves antigen-specific CD8^+^ T cell and proinflammatory Th1 responses. (**A**) Schematic overview of H2Db-restricted peptide pulsed syngeneic WT, MHC class I semiallogeneic bm1, or MHC class II semiallogeneic bm12 BMDC vaccine experiments in mouse models. (**B**) In vitro IFN-γ production of isolated CD8^+^ T cells from naive Pmel-1 mice stimulated with titrated LPS-matured BMDCs (mBMDCs) pulsed with or without gp100_25–33_ peptide by ELISA. Isolated splenic Pmel-1 CD8^+^ T cells (1 × 10^5^) were cocultured with different ratios of the WT, bm1, or bm12 mBMDCs with or without 20 μg/mL gp100_25–33_ peptide pulsing in 200 μL/well in 96-well round-bottom plates for 24 hours. mBMDCs, Pmel-1 CD8^+^ T cells, or hgp100 alone were controls. (**C**–**E**) WT mice were injected s.c. with 1 × 10^5^ B16-F10 tumor cells, and mice were injected intradermally (i.d.) with either of the gp100_25–33_-pulsed WT, bm1, or bm12 mBMDC vaccines 3 times starting 4 days after tumor inoculation at 5-day intervals. (**C**) Experimental scheme and (**D**) average and (**E**) individual tumor growth. The mice noted in **D** and **E** were sacrificed 14 days after B16-F10 tumor inoculation, and tumors were subjected to flow cytometric analysis of infiltrating CD8^+^ and hgp100/H2Db tetramer^+^ cells and CD4^+^ T cell subsets. Individual mouse values of (**F**) tumor weight, (**G**) hgp100/H2Db tetramer^+^CD69^+^CD8^+^ T cells, and (**H**) Th1 cell in tumors. Data are presented as mean ± SEM and represent 3 independent experiments, *n* = 3 (**B**), 4–5 (**D** and **E**) and 6–9 (**F**–**H**) per group. Statistical analysis was performed using 2-way (**D**) or 1-way (**E**, **F**, and **H**) ANOVA test with post hoc Tukey’s multiple-comparison correction. *P < 0.05; **P < 0.01; ***P < 0.001; ****P < 0.0001.

We evaluated therapeutic use of H2Db-restricted peptide–pulsed MHC class I or MHC class II semiallogeneic BM-derived DC (BMDC) vaccines compared to syngeneic BMDCs against tumors in murine models (Figure 1A). Syngeneic BMDCs were generated from C57BL/6J (WT) and semiallogeneic BMDCs were generated from 2 mouse strains, B6.C-*H2-K^bm1^*/ByJ (bm1), which carries mutations in H2Kb MHC class I (H2Kbm1), or B6(C)-*H2-Ab1^bm12^*/KhEgJ (bm12) that carries mutations in IAb MHC (IAbm12) (Table 1) (32). Before testing these BMDC vaccines in vivo, we compared characteristics of each BMDC in vitro. The WT, bm1, and bm12 BMDCs were generated by culturing BM cells from each strain with 20 ng/mL GM-CSF for 10 days. The WT, bm1, and bm12 BMDCs resembled monocyte-derived DCs with a surface CD11c^+^MHC II^+^CD11b^+^B220^−^CD8a^−^ phenotype and they matured similarly, showing greater than 95% of the population as CD45^+^CD11c^+^ cells and CD11c^+^MHC class II (I-A/I-E)^+^ (Supplemental Figure 1, A and B; supplemental material available online with this article; https://doi.org/10.1172/jci.insight.189024DS1). Furthermore, all 3 BMDCs expressed similar levels of MHC class I and II and costimulatory molecules after generation with GM-CSF from the BM cells. The BMDCs were also matured with 100 ng/mL LPS (labeled mBMDCs) or stimulated with WT splenocytes for 18 hours, with similar effects on each strain of BMDC on their expression of MHC and costimulatory molecules. Furthermore, WT splenocytes increased the expression of MHC class II and CD86 to a greater extent than LPS maturation alone (Supplemental Figure 1C). In a mixed lymphocyte reaction (MLR) of WT splenocytes cocultured with WT, bm1, or bm12 BMDCs, WT CD8^+^ T cells proliferated more when stimulated by the bm1 and bm12 BMDCs compared with WT BMDCs. WT CD4^+^ T cells proliferated more when stimulated by the bm12 BMDCs, resulting in an increase in total CD3^+^ T cells (Supplemental Figure 1, D and E). These results suggest that the MHC class II semiallogeneic bm12 BMDC vaccine had the potential to provide allogeneic CD4^+^ T cell help to promote tumor suppression in vivo.

### WT, bm1, and bm12 mature BMDCs present H2Db-restricted gp100_25–33_ peptide to CD8^+^ T cells similarly, but the bm12 BMDC vaccine is superior in delaying B16-F10 tumor growth and improves antigen-specific CD8^+^ T cell responses and proinflammatory Th1 responses.

To confirm whether the syngeneic WT, MHC class I semiallogeneic (bm1) and MHC class II semiallogeneic (bm12) BMDCs pulsed with H2Db-restricted peptide could present that antigen to CD8^+^ T cells in the same manner, we stimulated isolated T cell receptor–transgenic (TCR-transgenic) Pmel-1 CD8^+^ T cells with mBMDCs pulsed with human gp100_25–33_ (hgp100_25–33_) peptide (Figure 1B). Pmel-1 transgenic mice, which are on the C57BL/6J background, express a Vα1Vβ13 TCR specific for the H2Db-restricted epitope corresponding to gp100_25–33_ (33). This enables its recognition by Pmel-1 CD8^+^ T cells. B16-F10 is a melanoma cell line on the C57BL/6J background that expresses the mouse gp100 antigen (34). Although the hgp100_25–33_ and murine (mgp100_25–33_) epitopes are homologous, the 3 NH_2_-terminal amino acid differences increase the human peptide’s ability to stabilize “empty” H2Db and trigger more T cell IFN-γ production (35). Others have reported that immunization with xenogeneic hgp100_25–33_ in the mouse B16-F10 melanoma model is superior to immunization with syngeneic mgp100_25–33_ (36, 37). Thus, we used hgp100_25–33_ peptide to pulse mBMDC vaccines in this study. Titrated WT, bm1, and bm12 hgp100_25–33_-pulsed mBMDCs stimulated Pmel-1 CD8^+^ T cells, producing similar amounts of IFN-γ (Figure 1B). Purity of isolated Pmel-1 CD3^+^CD8^+^ T cells among CD45^+^ cells was greater than 90% in this assay (Supplemental Figure 2A). To determine the therapeutic effect of these 3 peptide-pulsed mBMDC vaccines, hgp100_25–33_-pulsed WT, bm1, or bm12 mBMDC vaccines were injected intradermally (i.d.) in B16-F10 tumor–bearing WT mice (Figure 1C). Both the hgp100_25–33_-pulsed WT and bm1 mBMDC vaccines showed significant efficacy compared with the PBS group but there was no significant difference between those 2 vaccines. However, the hgp100_25–33_-pulsed bm12 mBMDC vaccine showed significantly greater efficacy than the other 2 hgp100_25–33_ mBMDC vaccines in delaying B16-F10 tumor growth (Figure 1, D and E). Vaccination with hgp100_25–33_-pulsed bm12 mBMDCs resulted in decreased tumor weight and increased infiltrating hgp100/H2Db tetramer–specific CD69^+^CD8^+^ T cells 14 days after B16-F10 inoculation (Figure 1, F and G; tumor growth curves until the harvesting are shown in Supplemental Figure 3, A and B). In addition, CD4^+^ T cell subset analysis in tumor-infiltrating cells showed Th1 cells increased significantly more in the mice that received the hgp100_25–33_-pulsed bm12 mBMDCs than mice that received either the hgp100_25–33_-pulsed WT or bm1 mBMDC vaccines (Figure 1H), but levels of Th2 and Tfh cells and Tregs were unchanged (Supplemental Figure 3C). The hgp100_25–33_-pulsed bm12 mBMDC vaccine increased Th1 cells induced by the allogeneic MHC class II molecules, and that can improve the peptide-specific CD8^+^ T cell response compared with the other mBMDCs.

### The E7_43–77_-pulsed bm12 mBMDC vaccine is superior to WT and bm1 mBMDC vaccines in delaying TC-1 tumor growth and improves antigen-specific CD8^+^ T cell responses and proinflammatory Th1 and Th17 responses.

**Figure 2:**
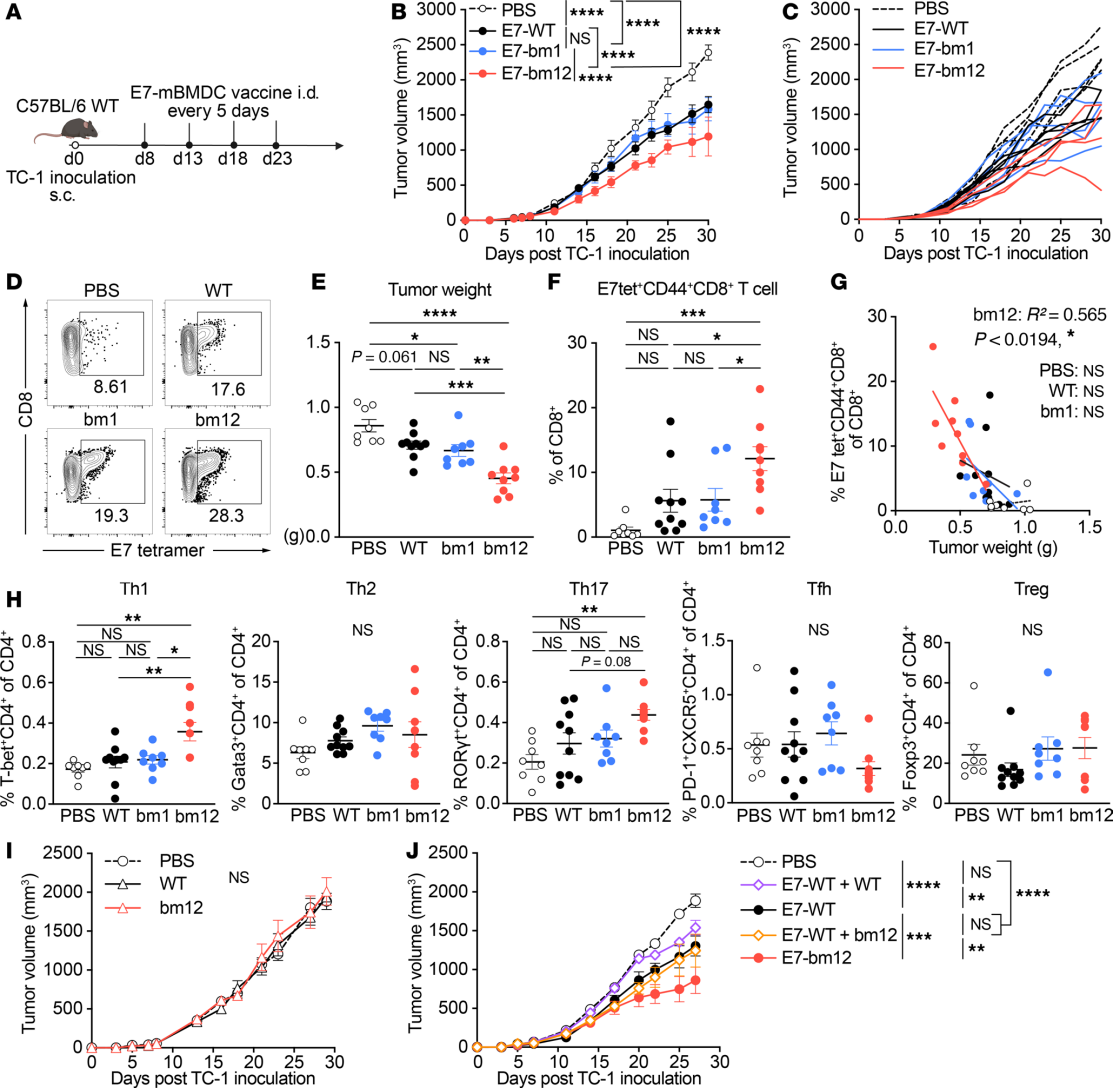
The E7_43–77_ peptide–pulsed MHC class II semiallogeneic mature BMDC vaccine is superior in delaying TC-1 tumor growth, improves the E7-specific CD8^+^ T cell, and increases proinflammatory Th1 and Th17 T cells. (**A**–**C**) WT mice were injected s.c. with 1 × 10^5^ TC-1 tumor cells and injected intradermally (i.d.) with either of the E7_43–77_ peptide–pulsed syngeneic WT, MHC class I semiallogeneic bm1, or MHC class II semiallogeneic bm12 LPS-matured BMDC (mBMDC) vaccines 4 times starting 8 days after tumor inoculation at 5-day intervals. (**A**) Experimental scheme. (**B**) Average and (**C**) individual tumor growth of each tumor-bearing mouse. (**D**–**H**) The mice noted in **B** and **C** were sacrificed 22 days after TC-1 tumor inoculation, and tumors were subjected to flow cytometric analysis of infiltrating CD8^+^ and E7/H2Db tetramer^+^ cells and CD4^+^ T cell subsets in tumors. (**D**) Representative flow cytometry contour plots for E7/H2Db tetramer^+^CD8^+^ T cells. Numbers in the plots indicate the percentage of gated cells. (**E**) Individual mouse values of tumor weight and (**F**) E7/H2Db tetramer^+^CD44^+^CD8^+^ T cells, and (**G**) correlation between tumor-infiltrating E7/H2Db tetramer^+^CD44^+^CD8^+^ T cells (*y* axis) and tumor weight (*x* axis) of indicated groups. (**H**) Individual mouse values of CD4^+^ T cell subsets. (**I** and **J**) WT mice were injected s.c. on the same schedule as **A** with 1 × 10^5^ TC-1 tumor cells and (**I**) WT or bm12 mBMDCs without peptide pulsing or (**J**) E7_43–77_ peptide–pulsed WT mBMDCs mixed with either WT or bm12 mBMDCs. Data are presented as mean ± SEM and represent 3 independent experiments, *n* = 4–5 (**B**, **C**, **I**, and **J**) and 8–10 (**E**–**H**) per group. Statistical analysis was performed using 2-way (**B**, **I**, and **J**) or 1-way ANOVA (**E**, **F**, and **H**) test with post hoc Tukey’s multiple-comparison correction, or linear regression analysis (**G**). *P < 0.05; **P < 0.01; ***P < 0.001; ****P < 0.0001.

To examine whether the superior efficacy of the peptide-pulsed bm12 BMDC vaccine was consistent across multiple tumor models, we next tested the therapeutic use of the peptide-pulsed WT, bm1, and bm12 mBMDC vaccines in the TC-1 tumor mouse model. TC-1 is a cancer cell line derived from primary lung epithelial cells of C57BL/6J mice and expresses HPV 16 oncogene proteins E6 and E7 (38, 39). Melief et al. have shown that the long E7_43–77_ peptide primes E7-specific CD4^+^ T helper cells as well as E7_49–57_-specific CD8^+^ T cells and results in better antitumor activity than the shorter E7_49–57_ peptide (40, 41). Therefore, we pulsed these mBMDCs with the long E7_43–77_ peptide. The E7_43–77_-pulsed WT, bm1, and bm12 mBMDC vaccines were injected i.d. into TC-1 tumor–bearing WT mice starting 8 days after TC-1 inoculation when tumors reached at least 5 mm in diameter and at least 40 mm^3^ in volume (Figure 2A). TC-1 tumor growth after these BMDC vaccinations showed results comparable to those in the B16-F10 model, such that the E7_43–77_-pulsed WT and bm1 mBMDC vaccines were able to reduce tumor growth compared with the PBS control group, but there was no significant difference between those 2 vaccines. However, the E7_43–77_-pulsed bm12 mBMDC vaccine showed efficacy significantly superior to the other 2 mBMDC vaccines (Figure 2, B and C). Vaccination with E7_43–77_-pulsed bm12 mBMDCs resulted in decreased tumor weight and increased infiltrating E7/H2Db tetramer^+^CD44^+^CD8^+^ T cells 22 days after TC-1 inoculation (Figure 2, D, F, and G; gating strategy, and Supplemental Figure 4). Tumor-infiltrating E7/H2Db tetramer^+^CD44^+^CD8^+^ T cells were plotted against tumor weight 22 days after TC-1 inoculation (Figure 2G), revealing a significant negative correlation (*R*^2^ = 0.565, *P* = 0.0194) between them only for the E7_43–77_-pulsed bm12 mBMDC vaccination group. In addition, CD4^+^ T cell subset analysis in tumor-infiltrating cells showed Th1 and Th17 cells increased significantly more in the mice that received the E7_43–77_-pulsed bm12 mBMDCs than mice that received either the E7_43–77_-pulsed WT or bm1 mBMDC vaccines, but levels of Th2 and Tfh cells and Tregs were unchanged (Figure 2H; gating strategy, and Supplemental Figure 4). The E7_43–77_-pulsed bm12 mBMDC vaccine generates CD4^+^ T cell subset changes induced by the allogeneic MHC class II molecules, and that can improve the peptide-specific CD8^+^ T cell response compared with the other mBMDCs.

These results raise the question whether such allogeneic help induced by E7_43–77_-pulsed bm12 mBMDCs might be effective at reducing tumor growth even without tumor-antigen pulsing. To that end, the WT or bm12 mBMDCs without E7_43–77_ peptide were injected in TC-1 tumor–bearing mice on the same schedule for the E7_43–77_-pulsed mBMDC vaccine (Figure 2A), but neither the unpulsed WT nor bm12 mBMDCs were effective for delaying tumor growth (Figure 2I). Then we tested whether the allo-MHC II could be on a different DC from the one presenting the peptide, by injecting either the unpulsed WT or bm12 mBMDCs mixed with the E7_43–77_-pulsed WT mBMDCs in TC-1 tumor–bearing mice. The mixed vaccine of the unpulsed bm12 mBMDCs and the E7_43–77_-pulsed WT mBMDCs showed similar efficacy for delaying tumor growth as the E7_43–77_-pulsed WT mBMDC vaccine, whereas the E7_43–77_-pulsed bm12 mBMDCs were more efficacious (Figure 2J). These results suggest that the peptide must be loaded directly on the semiallogeneic BMDCs to stimulate cytotoxic CD8^+^ T cells, and then the alloantigen-specific CD4^+^ T cell response can synergistically work to enhance CD8^+^ T cell suppression of tumor growth. In other words, the same DC that stimulates the allo-CD4^+^ T cell response should be the one also presenting peptide to CD8^+^ T cells, presumably because its antigen-presenting activity is increased by the allostimulation. From that perspective, the semiallogeneic mBMDC strategy should be superior to conventional WT DC cancer vaccines.

### E7_43–77_-pulsed bm12 mBMDCs generate allogeneic CD4^+^ T cell responses through allogeneic MHC class II to support E7 peptide–dependent CD8^+^ T cell responses in TC-1 tumor–bearing mice.

**Figure 3:**
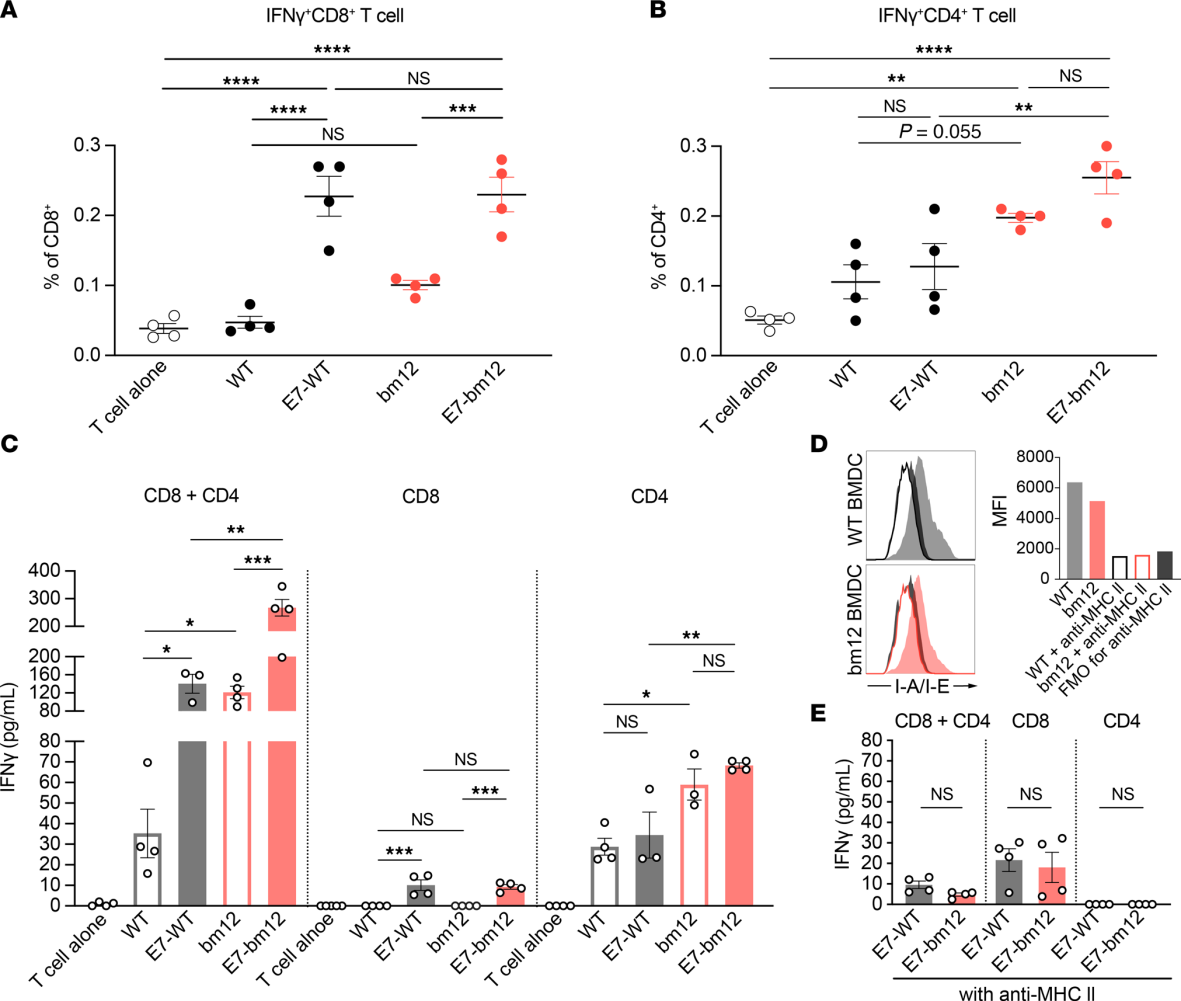
The E7_43–77_ peptide–pulsed MHC class II semiallogeneic mature BMDCs activate allogeneic CD4^+^ T cells through allogeneic MHC class II to support E7-dependent CD8^+^ T cell responses in TC-1 tumor–bearing mice. (**A** and **B**) Flow cytometric analysis of intracellular staining of IFN-γ in splenic CD8^+^ or CD4^+^ T cells stimulated with either syngeneic WT or MHC class II semiallogeneic bm12 mature BMDCs (mBMDCs) with or without E7 peptide pulsing. (**A**) CD8^+^ T cells and (**B**) CD4^+^ T cells were cocultured with indicated mBMDCs in 200 μL/well of 96-well round-bottom plates for 18 hours and then 6 hours with brefeldin A. (**C**) IFN-γ production of splenic CD8^+^ T cells and/or CD4^+^ T cells from TC-1 tumor–bearing mice stimulated with either WT or bm12 mBMDCs with or without peptide pulsing by ELISA. CD8^+^ T cells alone, CD4^+^ T cells alone, or a mixture of CD8^+^ T cells and CD4^+^ T cells were cocultured with indicated mBMDCs in 200 μL/well in 96-well round-bottom plates for 72 hours. (**D**) The WT or bm12 BMDCs blocked with anti–MHC class II antibody for 1 hour were subjected to flow cytometric analysis. The histograms indicate the change in I-A/I-E expression on each BMDC and the bar graphs indicate the median fluorescence intensity (MFI) of I-A/I-E with or without MHC II blocking and fluorescence minus one (FMO) control for the anti–MHC class II antibody. (**E**) Contrast values of IFN-γ production in the supernatants with those of **C** with MHC II blocked. In **A**–**C**, and **E**, 2 × 10^5^ isolated CD8^+^ T cells or 2 × 10^5^ isolated CD4^+^ T cells were cultured alone or mixed with 1 × 10^5^ indicated mBMDCs. Data are presented as the mean ± SEM and represent 3 independent experiments, *n* = 4–5 per group. Statistical analysis was performed using 1-way ANOVA test with post hoc Tukey’s multiple-comparison correction. *P < 0.05; **P < 0.01; ***P < 0.001; ****P < 0.0001.

To explore the mechanism of the MHC class II semiallogeneic mBMDCs in vitro, we measured IFN-γ–expressing CD8^+^ or CD4^+^ T cells from TC-1 tumor–bearing mice in cocultures of isolated splenic CD8^+^ T cells or CD4^+^ T cells stimulated for 24 hours with either WT or bm12 mBMDCs with or without E7_43–77_ pulsing (Figure 3, A and B). Purity of isolated CD8^+^ T or CD4^+^ T cells among CD45^+^ cells was greater than 90% (Supplemental Figure 2B). Both WT and bm12 E7_43–77_-pulsed mBMDCs stimulated CD8^+^ T cells to express IFN-γ, but neither the WT nor bm12 mBMDCs without E7_43–77_ pulsing stimulated IFN-γ expression (Figure 3A). On the other hand, the bm12 mBMDCs stimulated significantly more CD4^+^ T cells than the WT mBMDCs to express IFN-γ regardless of E7_43–77_ pulsing (Figure 3B). Next, we stimulated purified splenic CD8^+^ T cells or CD4^+^ T cells, or a mixture of the two, with either WT or bm12 mBMDCs with or without E7_43–77_ pulsing and measured total IFN-γ production in the supernatant after 72 hours (Figure 3C). The CD8^+^ T cell response was E7 dependent, because there was no IFN-γ detected when CD8^+^ T cells alone were stimulated with the unpulsed WT or bm12 mBMDCs (Figure 3C). On the other hand, the CD4^+^ T cell response was E7 independent, and the bm12 mBMDCs stimulated CD4^+^ T cells significantly better than the WT mBMDCs regardless of E7_43–77_ pulsing (Figure 3C). Mixtures of CD8^+^ and CD4^+^ T cells stimulated with the E7_43–77_-pulsed bm12 mBMDCs produced significantly more IFN-γ than in the other 3 coculture assays (Figure 3C). Moreover, the E7_43–77_-pulsed bm12 mBMDCs stimulated almost twice as much IFN-γ production as the E7_43–77_-pulsed WT mBMDCs. These results suggest that CD4^+^ T cells stimulated by the bm12 mBMDCs can strengthen the CD8^+^ T cell response directly by peptide loaded on the H2Db molecules on the bm12 mBMDCs. Next, we asked whether this allogeneic CD4^+^ T cell help was due to the mutant MHC class II, so we stimulated mixtures of CD8^+^ and CD4^+^ T cells, or CD8^+^ or CD4^+^ T cells alone, with the E7_43–77_-pulsed WT or bm12 mBMDCs that had MHC class II blocked beforehand (Figure 3E). We confirmed that the anti–MHC class II antibody blocked MHC class II molecules on both BMDCs by flow cytometry (Figure 3D). Neither WT nor bm12 mBMDCs in the presence of MHC class II blockade stimulated CD4^+^ T cells, and that resulted in much lower IFN-γ production in the coculture of mixed CD8^+^ and CD4^+^ T cells (Figure 3E). These results suggest allogeneic CD4^+^ T cell help generated by the bm12 mBMDCs is mutant MHC class II dependent.

### The E7_43–77_-pulsed bm12 mBMDCs expand Th1, Th2, Tfh, and Th17 cells but reduce the proportion of Tregs.

**Figure 4:**
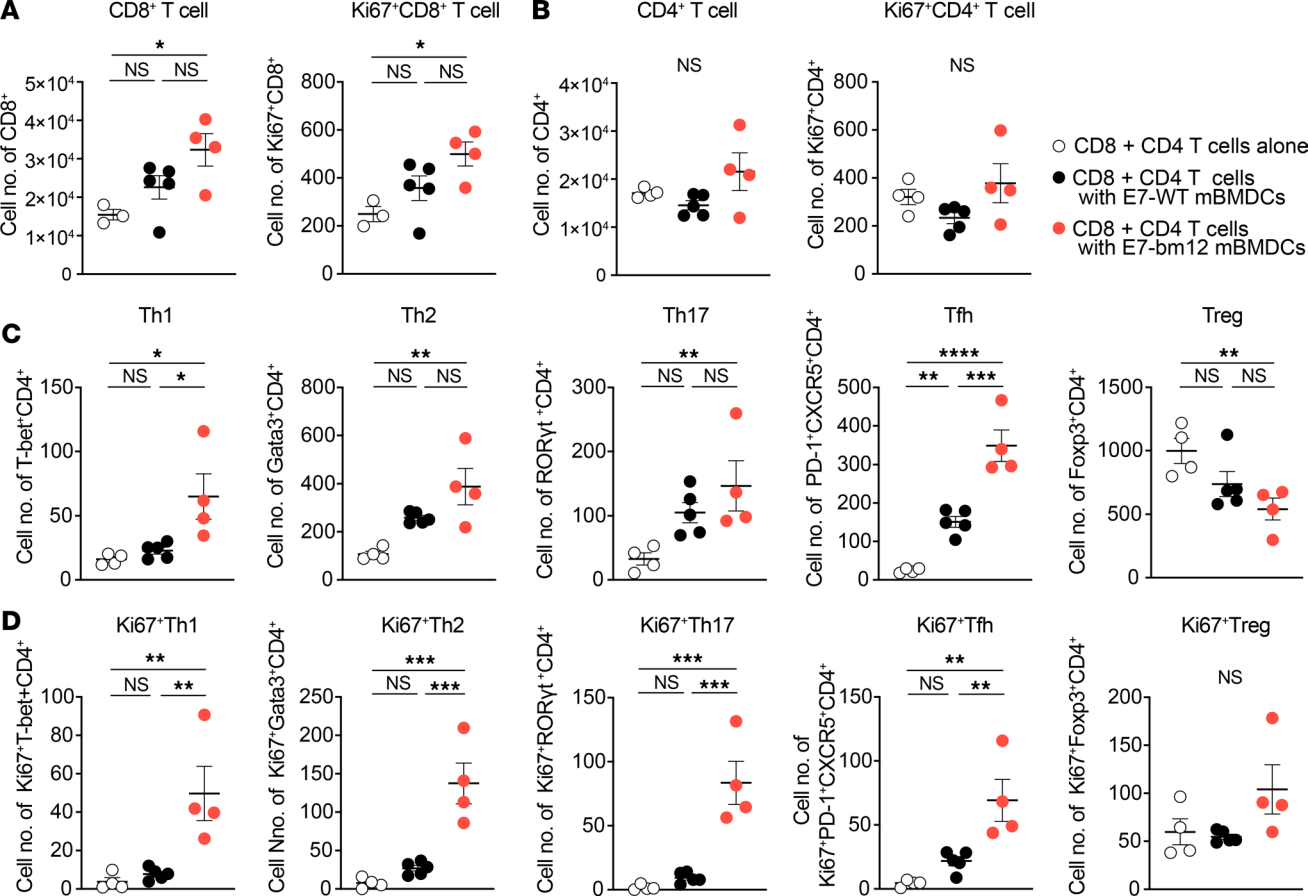
E7_43–77_ peptide–pulsed MHC class II semiallogeneic mature BMDCs expand Th1, Th2, Tfh, and Th17 cells but reduce the proportion of Tregs. (**A**–**D**) In vitro CD8^+^ or CD4^+^ T cell subset changes after coculture of mixtures of splenic CD8^+^ T cells and CD4^+^ T cells from TC-1 tumor–bearing mice stimulated with either the E7_43–77_ -pulsed syngeneic WT or MHC class II semiallogeneic bm12 mBMDCs. TC-1 tumor–bearing mice were sacrificed 8–12 days after 1 × 10^5^ TC-1 inoculation and mixtures of 2 × 10^5^ isolated CD8^+^ T cells and 2 × 10^5^ isolated CD4^+^ T cells were cocultured with 1 × 10^5^ cells of indicated mBMDCs in 200 μL/well in 96-well round-bottom plates for 48 hours and subjected to flow cytometric analysis. Data are presented as the mean ± SEM and represent 2 independent experiments, *n* = 4–5 per group. Statistical analysis was performed using 1-way ANOVA test with post hoc Tukey’s multiple-comparison correction. *P < 0.05; **P < 0.01; ***P < 0.001; ****P < 0.0001. mBMDC, LPS-matured BMDC; SEM, standard error of mean; ANOVA, analysis of variance.

To reveal which CD4^+^ T cell subsets contribute to the allogeneic CD4^+^ T cell help that supports CD8^+^ T cell activation, we stimulated a mixture of splenic CD8^+^ and CD4^+^ T cells from TC-1 tumor–bearing mice with the E7_43–77_-pulsed mBMDCs of either the WT or bm12 strains. The final cell number of CD8^+^ T cells and the final cell number of Ki67-expressing cells as a marker of proliferating CD8^+^ T cells was significantly greater than the unstimulated control after 48 hours of coculturing in vitro only when they were stimulated with the E7_43–77_-pulsed bm12 mBMDCs (Figure 4A). Although there was no significant change in either the final cell number of CD4^+^ T cells or Ki67-expressing CD4^+^ T cells when they were stimulated with the E7_43–77_-pulsed WT or bm12 BMDCs (Figure 4B), the CD4^+^ T cell subsets changed remarkably after stimulation with the E7_43–77_-pulsed bm12 mBMDCs (Figure 4, C and D); Th1, Th2, Th17, and Tfh cells increased, but Tregs decreased significantly more after stimulation with the E7_43–77_-pulsed bm12 mBMDCs than with the E7_43–77_-pulsed WT mBMDCs (Figure 4C). The Tfh cells increased significantly with both types of stimulation. Ki67-expressing proliferating Th1, Th2, Th17, and Tfh cells were also significantly higher after stimulation with E7_43–77_-pulsed bm12 mBMDCs than those with WT mBMDCs, but Ki67-expressing proliferating Tregs were not significantly different among these groups (Figure 4D). We observed similar results from coculture of a mixture of CD8^+^ T cells and CD4^+^ T cells and either WT or bm12 mBMDCs without E7_43–77_ pulsing (Supplemental Figure 5), suggesting that the bm12 mutation was the dominant CD4 antigen, rather than E7, in contrast with the CD8^+^ T cells.

### The E7_43–77_-pulsed bm12 mBMDC vaccine is dependent on both CD8^+^ and CD4^+^ T cells, but CD4^+^ T cell depletion at the later stage of TC-1 tumor growth enhances the efficacy.

**Figure 5:**
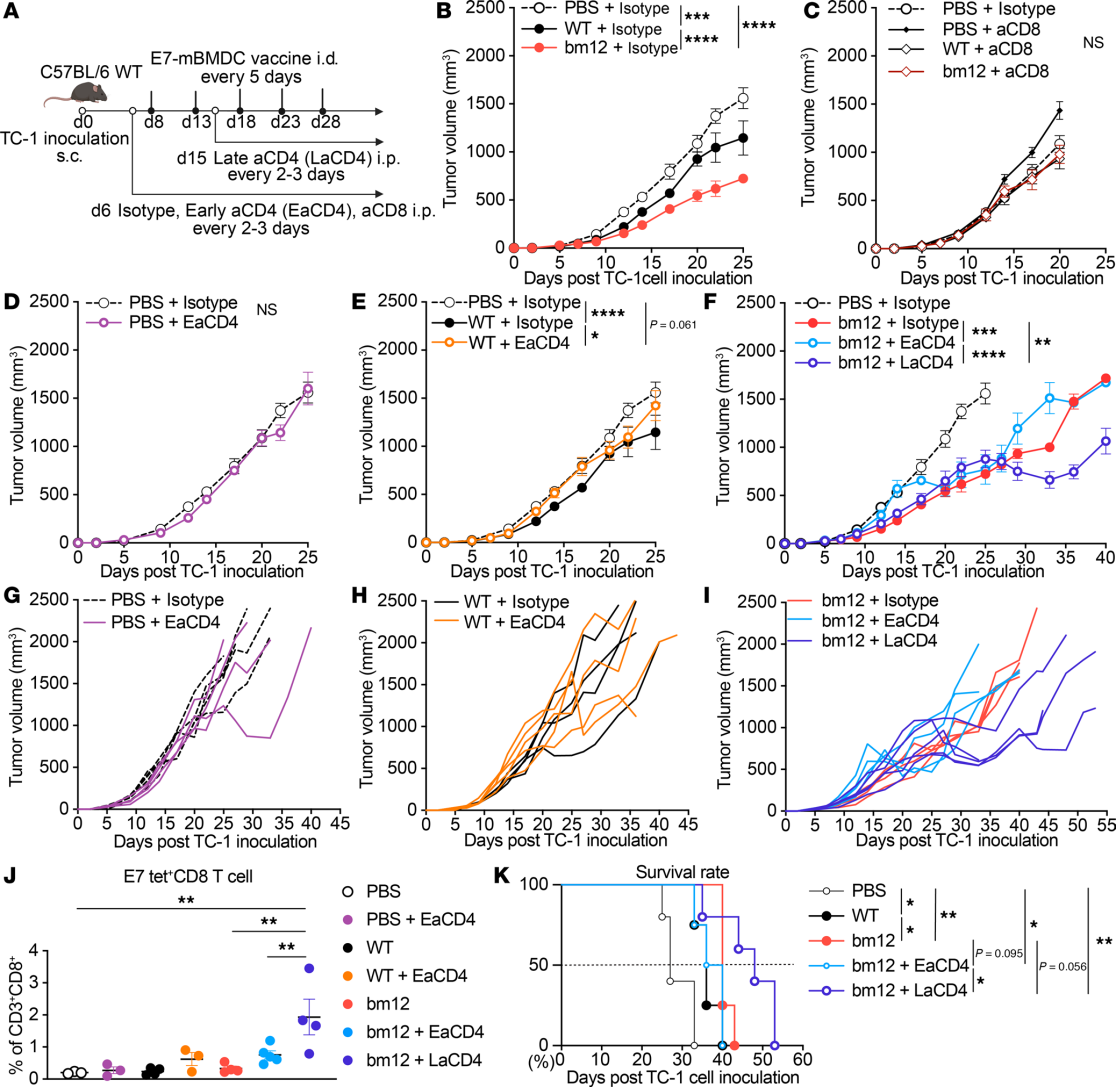
E7_43–77_-pulsed MHC class II semiallogeneic mature BMDC vaccination is dependent on both CD8^+^ and CD4^+^ T cells, but CD4^+^ T cell depletion at a later stage of TC-1 tumor growth enhances the efficacy. (**A**–**I**) WT mice were injected s.c. with 1 × 10^5^ TC-1 tumor cells, and mice were injected intradermally (i.d.) with either of the E7_43–77_-pulsed syngeneic WT or MHC class II semiallogeneic bm12 LPS-matured BMDC (mBMDC) vaccines 5 times starting 8 days after tumor inoculation at 5-day intervals. CD8^+^ or CD4^+^ T cells were depleted by i.p. injection of anti-CD4 antibody (clone GK1.5), anti-CD8 antibody (clone 53-6.7), or isotype control antibody (IgG) that started 2 days before the first dose of the BMDC vaccination for early depletion or that started 2 days after the second dose of BMDC vaccination for late depletion, and continued every 2–3 days until end of the study. (**A**) Experimental scheme, (**B**–**F**) average tumor growth of indicated groups, and (**G**–**I**) individual tumor growth of each tumor-bearing mouse. (**J**) Mouse blood was drawn 29 days after TC-1 inoculation in the same mice used in **B**–**F** and subjected to flow cytometric analysis of circulating CD8^+^ and E7/H2Db tetramer^+^ cells in blood. (**K**) Survival rate of indicated groups. The median value of each group: PBS, 27 days; WT, 36 days; bm12, 40 days; bm12 + EaCD4, 38 days; bm12 + LaCD4, 48 days. Data are presented as the mean ± SEM and represent 3 independent experiments, *n* = 4–5 per group. Statistical analysis was performed using 2-way ANOVA over the entirety of the study (**B**–**F**), 1-way ANOVA test with post hoc Tukey’s multiple-comparison correction (**J**), or Kaplan-Meier curves and analyzed by log-rank (**K**). *P < 0.05; **P < 0.01; ***P < 0.001; ****P < 0.0001.

To determine whether the E7_43–77_-pulsed bm12 mBMDC vaccine response is dependent on CD8^+^ T cells through CD4^+^ T cell help in vivo, we selectively depleted CD8^+^ T cells starting 2 days before the first dose of the BMDC vaccine until the end of the study, or depleted CD4^+^ T cells on the same schedule as the depletion for CD8^+^ T cells as “Early CD4 depletion (EaCD4)” in mice receiving either the E7_43–77_-pulsed WT or bm12 mBMDC vaccines. Mice receiving the E7_43–77_-pulsed bm12 mBMDC vaccines were additionally depleted of CD4^+^ T cells starting 2 days after the second dose of the BMDC vaccination to the end of the study as “Late CD4 depletion (LaCD4)” (Figure 5A). CD4^+^ or CD8^+^ T cell depletion was confirmed using blood samples from those mice 7, 15, and 29 days after TC-1 inoculation (Supplemental Figure 6). Isotype antibody (IgG) controls did not influence the efficacy of mBMDC vaccines, but CD8^+^ T cell depletion abrogated the efficacy of both vaccines, indicating that the peptide-pulsed mBMDC vaccines were CD8^+^ T cell dependent (Figure 5, B and C). Neither CD8^+^ nor CD4^+^ T cell depletion affected the tumor growth in the PBS controls without vaccine (Figure 5, C, D, and G). On the other hand, early CD4^+^ T cell depletion combined with the E7_43–77_-pulsed WT mBMDC vaccine abrogated its efficacy compared with isotype control, suggesting the E7_43–77_-pulsed WT mBMDC vaccine is CD4^+^ T cell dependent (Figure 5, E and H). However, we observed interesting results from combined CD4^+^ T cell depletion and the E7_43–77_-pulsed bm12 mBMDC vaccination. Early CD4^+^ T cell depletion abrogated the efficacy of the E7_43–77_-pulsed bm12 mBDMC vaccination initially until 17 days after TC-1 inoculation, and there was a significant difference between the vaccine with and without CD4^+^ T cell depletion at that early phase (*P* < 0.001). After that time point, the early CD4^+^ T cell depletion group recovered the efficacy of the E7_43–77_-pulsed bm12 mBMDC vaccine until the end of vaccination, whereas late CD4^+^ T cell depletion enhanced the efficacy of the E7_43–77_-pulsed bm12 mBMDC vaccine even after the end of the fifth dose of the vaccination. There was a marked significant difference between the bm12 DC vaccine alone and the PBS group (*P* < 0.0001) and the bm12 DC vaccine combined with late CD4^+^ T cell depletion and the PBS group (*P* < 0.0001) over the entirety of the tumor growth before the mice in the PBS group succumbed (on day 25 after TC-1 inoculation). Moreover, there was a significant difference between the bm12 DC vaccine alone and the vaccine with late CD4^+^ T cell depletion on day 40 after TC-1 inoculation (*P* < 0.01; Figure 5, F and I). Circulating E7-specific CD8^+^ T cells in blood 29 days after TC-1 inoculation were significantly higher in the mice that received late CD4^+^ T cell depletion combined with the E7_43–77_-pulsed bm12 mBMDC vaccine (Figure 5J), and that combination also prolonged the survival rate (Figure 5K). These results suggests that the MHC class II semiallogeneic bm12 mBMDC vaccination is CD4^+^ T cell dependent only at the early stages of the tumor growth, but depletion of CD4^+^ T cells during the later stages of tumor growth and treatment enhances mBMDC-mediated tumor suppression through improving E7-specific CD8^+^ T cell responses. We hypothesized that Tregs were the causative CD4^+^ T cell subset inhibiting the vaccine-induced response at later time points.

### Late Treg depletion enhances the efficacy of the E7_43–77_-pulsed bm12 mBMDC vaccine, maintaining intratumoral E7_43–77_-specific CD8^+^ T cell antitumor activity and increasing circulating E7-specific CD8^+^ T cells in blood.

**Figure 6:**
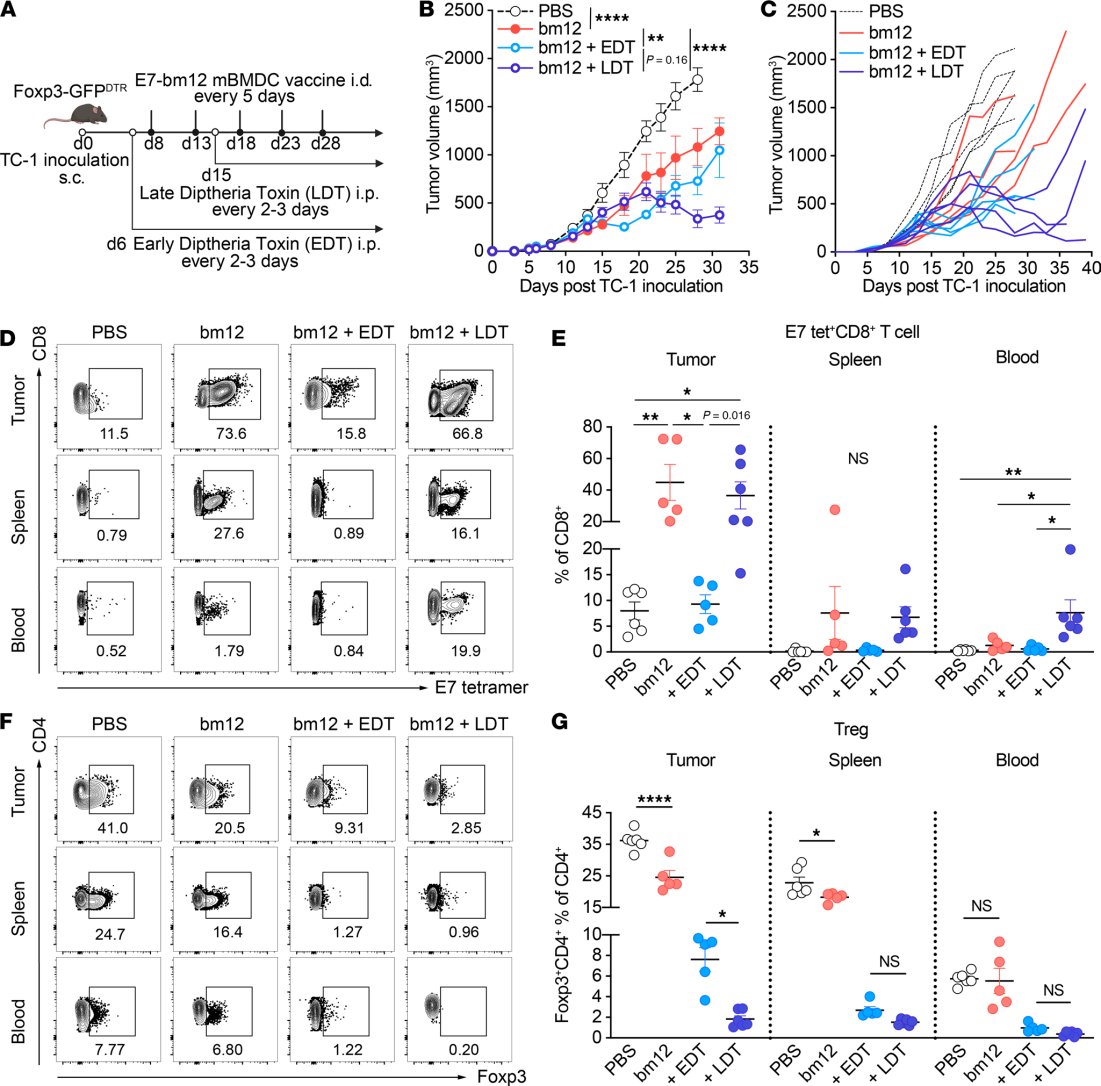
Late Treg depletion enhances the efficacy of the E7_43–77_-pulsed MHC class II semiallogeneic mature BMDC vaccination by maintaining intratumoral E7-specific CD8^+^ T cell antitumor activity and increasing circulating E7-specific CD8^+^ T cells in the blood. (**A**) Foxp3-GFP^DTR^ mice were injected s.c. with 1 × 10^5^ TC-1 tumor cells, and mice were injected intradermally (i.d.) with E7_43–77_-pulsed MHC class II semiallogeneic bm12 LPS-matured BMDC (mBMDC) vaccines (designated bm12 in the graphs) 5 times starting 8 days after tumor inoculation at 5-day intervals. Tregs were depleted by i.p. injection of diphtheria toxin (DT) that started 2 days before the TC-1 inoculation for early depletion (EDT) or 2 days after the second dose of the BMDC vaccinations for late depletion (LDT) and continued every 2–3 days until end of the study. (**A**) Experimental scheme, (**B**) average tumor growth of indicated groups, and (**C**) individual tumor growth of each tumor-bearing mouse. (**D**–**G**) Mice were sacrificed 23 days after TC-1 inoculation and subjected to flow cytometric analysis of CD8^+^ and E7/H2Db tetramer^+^ cells and Foxp3^+^ and CD4^+^ T cells in tumors, spleen, and blood. (**D** and **F**) Representative flow cytometry contour plots for indicated cells. (**E** and **G**) Individual mouse values of CD8^+^ and E7/H2Db tetramer^+^ cells (**E**) and Foxp3^+^ Tregs (**G**). Numbers in the plots indicate the percentage of gated cells. Data are presented as the mean ± SEM and represent 2 independent experiments, *n* = 5–6 per group (**B**–**G**). Statistical analysis was performed using 2-way ANOVA over the entirety of the study (**B**) and 1-way ANOVA with post hoc Tukey’s multiple-comparison correction (**E** and **G**). *P < 0.05; **P < 0.01; ****P < 0.0001.

To determine whether depleting Foxp3^+^ Tregs starting at the later stage of the tumor growth enhanced the antitumor activity of the MHC class II semiallogeneic bm12 mBMDC vaccine, we used B6.129(Cg)-*Foxp3^tm3(Hbegf/GFP)Ayr^*/J (Foxp3-GFP^DTR^) mice, which undergo selective depletion of Foxp3^+^ cells upon administration of diphtheria toxin (DT) (42). We began depletion of Tregs 2 days before the first dose of the E7_43–77_-pulsed bm12 mBMDC vaccine and continued until the end of the study as “early Treg depletion” (early DT injection, labeled EDT) or started depletion of Tregs toward the end of the study as “late Treg depletion” (late DT injection, labeled LDT) by intraperitoneal injection of DT every 2–3 days starting on day 15 (43). Then we injected the E7_43–77_-pulsed bm12 mBMDC vaccine in TC-1–bearing Foxp3-GFP^DTR^ mice every 5 days starting on day 8 after TC-1 inoculation (Figure 6A). Foxp3^+^ Treg depletion was confirmed using blood samples from those mice 9 days after TC-1 inoculation (Supplemental Figure 7). In contrast with early CD4^+^ T cell depletion combined with the E7_43–77_-pulsed bm12 mBMDC vaccine, early Treg depletion did not hamper bm12 mBMDC efficacy at the earlier stages of tumor growth, and tumor growth was delayed until 17 days after TC-1 inoculation, but gradually the tumors grew at a similar rate as those in the mice that received vaccination alone (Figure 6, B and C). However, late Treg depletion enhanced the E7_43–77_-pulsed bm12 mBMDC vaccine efficacy and the tumors started shrinking around 20 days after TC-1 inoculation, not just slowing down (Figure 6, B and C). Tumors in the mice that received the combination of late Treg depletion and the E7_43–77_-pulsed bm12 mBMDC vaccine kept shrinking around 30 days after TC-1 inoculation when the experiment was terminated (Figure 6C). We confirmed that the late Treg depletion alone did not suppress the TC-1 tumor growth, showing similar tumor growth with the PBS control group (Supplemental Figure 8). The tumor growth curves of the combinations with early and late Treg depletion crossed at 23 days after TC-1 inoculation, so we analyzed the percentage of intratumoral E7_43–77_-specific CD8^+^ T cells and Foxp3^+^ Tregs, as well as those in spleen and blood, 23 days after TC-1 inoculation when there was no difference in tumor size to affect the results (Figure 6, D–G; gating strategy, and Supplemental Figure 9). Tumor-infiltrating E7/H2Db tetramer^+^CD8^+^ T cells were significantly higher in the groups that received the E7_43–77_-pulsed bm12 mBMDC vaccine alone and in combination with late Treg depletion, but only the combination of vaccine with late Treg depletion significantly increased circulating E7/H2Db tetramer^+^CD8^+^ T cells in the blood (Figure 6, D and E), showing comparable results with those of the combination of the E7_43–77_-pulsed bm12 mBMDC vaccine and late CD4^+^ T cell depletion in blood 29 days after TC-1 inoculation (Figure 5J and Figure 6E). Late Treg depletion maintained high circulating E7/H2Db tetramer^+^CD8^+^ T cells in blood up to 29 days after TC-1 inoculation (Supplemental Figure 10A). We analyzed Foxp3^+^ Tregs on day 23 after TC-1 inoculation, showing the E7_43–77_-pulsed bm12 mBMDC vaccine alone slightly reduced the percentage of Foxp3^+^ Tregs in tumors and spleens compared with those in PBS group (Figure 6, F and G). The mice with early Treg depletion had significantly higher tumoral Foxp3^+^ Tregs on day 23 compared with those with late Treg depletion even though they had continuous administration of DT, and the tumor sizes were the same (Figure 6, F and G). The percentage of Tregs in the blood was plotted at different time points, and it gradually increased in all groups except for the mice that received late DT injection (Supplemental Figure 10B). This result suggested that the mice were becoming immune to DT and were possibly making antibodies against DT that muted its effect and allowing Tregs to recover. To test this hypothesis, we measured anti-DT IgG by ELISA in both groups of DT-treated mice. The early-DT mice produced anti-DT IgG by 8 days after starting DT injection (14 days after TC-1 inoculation) and they maintained that level for 17 days after starting DT injection (23 days after TC-1 inoculation) (Supplemental Figure 10C). Similarly, the late-DT mice, which did not start receiving DT until day 15 after tumor inoculation, did not develop anti-DT IgG antibodies until 8 days later, on day 23, although the exact time when anti-DT IgG began to appear is uncertain. These results support the hypothesis that greater reduction in intratumoral Tregs and enhanced antitumor efficacy in the late-DT group was due to the later appearance of anti-DT antibodies. The bm12 BMDC vaccination combined with late Treg depletion also prolonged the survival rate compared with vaccine alone (Supplemental Figure 10D).

## Discussion

In this study, we demonstrated that the antigen-pulsed MHC class II semiallogeneic BMDC vaccine was superior to the antigen-pulsed syngeneic or MHC class I semiallogeneic BMDC vaccine in delaying the growth of 2 different mouse tumors with distinct immunologic and oncologic features, B16-F10 and TC-1. These DCs stimulated H2Db-restricted CD8^+^ T cells comparably in vitro, but only MHC class II semiallogeneic DCs enhanced antitumor immunity through CD4^+^ T cell help in vivo. This help was driven by alloreactive CD4^+^ Th cells recognizing mutant MHC class II molecules, resulting in the expansion of effective CD4^+^ Th cells and reduction in Tregs in the TC-1 tumor model in vivo and vitro. Interestingly, late-stage CD4^+^ T cell depletion after vaccination led to tumor shrinkage, suggesting that Tregs were the primary depleted subset. Combining selective late Treg depletion and the peptide-pulsed MHC class II semiallogeneic BMDC vaccine enhanced the efficacy of tumor suppression and caused actual complete regression of tumors in vivo.

Previous studies using semiallogeneic hybrid vaccines highlighted the benefit of alloreactive CD4^+^ T cell help (23–31) but did not directly compare MHC class I and II mismatch strategies or clarify the role of shared APCs presenting both signals. Our model demonstrated that DCs presenting tumor antigen via MHC class I and simultaneously stimulating alloreactive CD4^+^ T cells via mutant MHC class II achieved superior CTL responses. Other recent reports on HLA-matched allogeneic DCs (44–49) emphasized feasibility and clinical activity but lacked mechanistic insight into CD4^+^ T cell–mediated enhancement. Our findings clarify this mechanism and underscore the translational potential of MHC class II semiallogeneic DCs as scalable, effective cancer vaccines.

To our knowledge, this study uniquely demonstrated that a peptide-pulsed semiallogeneic DC vaccine can enhance the direct CD8^+^ CTL response by presenting tumor-specific peptides on syngeneic MHC class I molecules, while also generating robust allospecific CD4^+^ T cell help via mutant MHC class II molecules. We clearly revealed that the allogeneic MHC class II molecules were responsible for the improved antitumor response compared with a syngeneic DC vaccine and demonstrated that the CD4^+^ T cell–dependent MHC class II semiallogeneic DCs enhanced CD8^+^ CTL activity in vivo and in vitro.

We first demonstrated that the peptide-pulsed semiallogeneic bm12 mBMDC vaccine was superior to WT and bm1 mBMDC vaccines in controlling B16-F10 tumors (Figure 1, D and E), despite this tumor’s poor immunogenicity (50, 51). The efficacious hgp100_25–33_-pulsed bm12 mBMDC vaccine increased tumor-infiltrating hgp100/H2Db tetramer^+^ active CD8^+^ T cells and Th1 cells in tumors (Figure 1, F–H), suggesting improved cooperation between CD4^+^ and CD8^+^ T cells. To further evaluate antigen-specific CD8^+^ T cell responses, we used the TC-1 model, which allowed longer tumor monitoring and assessment of E7/H2Db tetramer^+^CD8^+^ T cells (Figures 2, 5, and 6). Similarly, the efficacious E7_43–77_-pulsed bm12 mBMDC enhanced infiltration of E7/H2Db tetramer^+^ memory CD8^+^ T cells, correlating with tumor suppression, and this also correlated inversely with tumor weight at the termination of these experiments (Figure 2, D–G). That suggested the superior tumor suppression was brought about by antigen-specific CD8^+^ CTLs enhanced by CD4^+^ T cell help from the E7_43–77_-pulsed bm12 mBMDC vaccine. Importantly, peptide pulsing was essential, as alloantigens alone were insufficient to confer protection (Figure 2I). The observation that Th1 and Th17 cells were higher in tumor-infiltrating cells during TC-1 growth (Figure 2H) implied that the E7_43–77_-pulsed bm12 mBMDC vaccine induced allogeneic CD4^+^ T cell help.

We explored the role of CD4^+^ T cell subsets in the TC-1 model. CD4^+^ T cell depletion at early time points abrogated vaccine efficacy, whereas late depletion aided E7_43–77_-pulsed mBMDC vaccine efficacy at later time points and enhanced tumor control and CD8^+^ T cell responses (Figure 5, F, I, and J). In the studies where we depleted Foxp3^+^ Tregs, we used Foxp3-GFP^DTR^ transgenic mice and confirmed that the late depletion of Foxp3^+^ Tregs alone was ineffective (Supplemental Figure 8), but when combined with the E7_43–77_-pulsed bm12 mBMDC vaccine, it significantly enhanced tumor suppression and enhanced the frequency of E7/H2Db tetramer^+^CD8^+^ T cells especially in tumors (Figure 6, B–E). These data were supported by an earlier report by Yu et al., showing that late-stage CD25^+^ T cell depletion in tumors enhances antitumor immunity by relieving suppression of CD8^+^ T cell proliferation and IFN-γ production (52). Compared with CD25, Foxp3 is a more reliable Treg marker, as CD25 is also expressed on activated effector T cells, and some Foxp3^+^ Tregs lack CD25 (53, 54). However, the Foxp3-DTR system has limitations. DT administration in healthy adult Foxp3-GFP^DTR^ mice can induce autoimmune inflammation and mortality within 3 weeks (42, 55). Indeed, in our study, early Treg depletion failed to induce robust CD8^+^ T cell responses (Figure 6, D and E), possibly because the mice could not generate sufficient numbers or quality of CD8^+^ T cells due to such autoimmune disorders, or reduced Treg numbers at early stages of vaccination may have allowed for the proliferation of suboptimal CD8^+^ T cell clones that would otherwise be pruned. Additionally, anti-DT IgG responses were detected from day 8 after DT injection (Supplemental Figure 10C), which may have reduced DT efficacy and could explain suboptimal tumor control when both early and late depletion were combined (Figure 6, B and C). Tregs are known to suppress antitumor immunity and correlate with poor prognosis in various cancers (55). Although selective depletion or controlling Treg function has failed in the clinic (56), combining the MHC class II semiallogeneic DC vaccine with selective Treg depletion could represent a promising future therapeutic approach.

In conclusion, our in vivo and in vitro studies demonstrated that antigen-pulsed MHC class II semiallogeneic DC vaccines effectively enhanced CD4^+^ T cell help via allogeneic MHC class II, thereby promoting the generation of antigen-specific CD8^+^ CTLs. By designing DCs to present tumor antigens on syngeneic MHC class I and provide allo-CD4^+^ T cell help through mismatched MHC class II, we achieved potent coordination between CTLs and helper T cells to reinforce antitumor immunity. Notably, these effects were achieved using a single peptide-MHC complex, highlighting the efficiency of this approach.

Our work introduced a unique approach, to our knowledge, by employing MHC class II semiallogeneic DCs that also express syngeneic MHC class I molecules. Rather than using WT allogeneic DCs or tumor fusion cells, we engineered DCs to selectively express mutant allogeneic MHC class II molecules while preserving syngeneic MHC class I expression. This precise configuration enables controlled activation of CD4^+^ T cells via indirect allorecognition without eliciting overt allogeneic rejection. Unlike classical allogeneic DC vaccines, our engineered DCs do not trigger robust direct alloreactive CD8^+^ T cell responses, minimizing the risk of nonspecific immune activation. Additionally, our semiallogeneic DC vaccine approach suggests potential strategies for human application. We propose that DC vaccines derived from semiallogeneic healthy donors are a more practical and efficacious approach for inducing an antitumor response than autologous DC vaccines. Moreover, large-scale generation of such semiallogeneic DCs from a pool of donors or engineered DC cell lines using MHC editing could enable an “off-the-shelf” solution for broad patient applicability. To mimic our murine strategy in humans, one potential approach involves CRISPR/Cas9-mediated editing of human MHC genes to generate DCs that express a selected MHC class I allele and a mismatched or mutated MHC class II molecule (such as HLA-DR or HLA-DQ) that can broadly stimulate allo-CD4^+^ T cell help. Importantly, while DC vaccines do not carry contaminating donor T cells and therefore do not cause classical graft-versus-host disease (GVHD), the use of allogeneic MHC class II can lead to excessive host T cell activation, posing a risk of GVHD-like inflammatory responses. To mitigate this, future studies could explore restricting expression to selected MHC class II molecules that enhance CD4^+^ T cell help without provoking detrimental host immune responses. The feasibility of ex vivo validation using HLA-mismatched human T cells and engineered DCs will be a critical step for clinical translation. Collectively, our findings offer a strong rationale for developing universal donor DC vaccines through MHC engineering and immune modulation, potentially transforming the landscape of cancer immunotherapy.

## Methods

### Sex as a biological variable.

Our study examined female mice because female animals exhibit less variability in phenotype. It is unknown whether the findings are relevant for male mice.

### Mice.

Female C57BL/6J (WT), B6.C-*H2-K^bm1^*/ByJ (bm1), B6(C)-*H2-Ab1^bm12^*/KhEgJ (bm12), B6.129(Cg)-*Foxp3^tm3^(Hbegf/GFP)Ayr*/J (Foxp3-GFP^DTR^), and B6.Cg-*Thy1^a^*/Cy Tg(TcraTcrb)8Rest/J (Pmel-1) transgenic mice were purchased from The Jackson Laboratory. All mice were maintained at the National Cancer Institute, NIH animal facility, and Pmel-1 transgenic, bm1, and bm12 mice were in-house bred at the NCI, NIH animal facility. WT mice and Foxp3-GFP^DTR^ transgenic mice aged 8–10 weeks old were used for tumor inoculation. WT, bm1, and bm12 mice aged 8–20 weeks old were used for generating BMDCs. Pmel-1 transgenic mice aged 8–12 weeks old were used for isolating splenocytes. All mice were handled on an Animal Study Protocol approved by the NCI Animal Care and Use Committee in a specific pathogen–free and Helicobacter-free AAALAC-approved animal facility.

### BMDCs.

To generated BMDCs, we used protocols reported by others (57). BM cells were isolated from WT, bm1, and bm12 mice suspended at a concentration of 2 × 10^5^ cells/mL in complete RPMI 1640 medium supplemented with 10% FCS, L-glutamine, sodium pyruvate, nonessential amino acids, streptomycin, and penicillin (cRPMI) in the presence of 20 ng/mL GM-CSF (Peprotech) for 10 days at 37°C and 5% CO_2_. Fresh cRPMI supplemented with 20 ng/mL GM-CSF was added on day 3 and refreshed on day 6 and day 8. Cells were harvested on day 10 and frozen until use. The resulting DCs were CD11c^+^MHC II^+^CD11b^+^B220^–^CD8a^–^ (Supplemental Figure 1).

### Peptide-pulsed mature BMDC preparation.

Frozen BMDCs were thawed and suspended at a concentration of 1 × 10^6^ cells/mL in cRPMI. To prepare mature BMDCs, BMDCs were incubated with 100 ng/mL LPS for 18–24 hours at 37°C and 5% CO_2_. To prepare antigen-pulsed mBMDCs, BMDCs were pulsed with 10 μg/mL peptide (gp100_25–33_, MedChemExpress or E7_43–77_, United Biosystems) for 1–2 hours, and then the cells were induced to mature by the addition of 100 ng/mL LPS for 18–24 hours at 37°C and 5% CO_2_. Cells were harvested by adding 2 mM EDTA (Invitrogen) for 15 minutes on ice followed by use of a cell scraper and washed twice with PBS and suspended at a concentration of 20 × 10^6^ cells/mL in PBS.

### MLR.

To assess proliferation of WT splenocytes with the WT, bm1, and bm12 BMDCs, WT splenocytes from naive WT mice were suspended at 1 × 10^6^ cells/mL in cRPMI and were stained with 0.1 mM CFSE (Invitrogen) for 20 minutes in a 37°C water bath in the dark. The stained splenocytes were washed twice with PBS, then 4 × 10^5^ CFSE-stained WT splenocytes were cultured alone or cocultured with 1 × 10^5^ cells of the WT, bm1, or bm12 BMDCs in 200 μL/well in 96-well round-bottom plates for 5 days. Gated CD3^+^ T cells, CD8^+^ T cells, and CD4^+^ T cells were analyzed for the dilution of CFSE by flow cytometry. To assess MHC and costimulatory molecules on BMDCs or mBMDCs, 1 × 10^5^ WT, bm1, or bm12 BMDCs or mBMDCs were cultured alone or cocultured with 4 × 10^5^ WT splenocytes in 200 μL/well in 96-well round-bottom plates for 18 hours. The antibodies used for staining cells in the MLR are listed in Supplemental Table 1.

### Mouse tumor studies.

The B16-F10 melanoma cell line was obtained from the American Type Culture Collection (ATCC, CRL-6475) and cultured in cRPMI at 37°C and 5% CO_2_. The TC-1 tumor cell line, a C57BL/6J-derived lung epithelial cell line transfected with HPV16 E6 and E7 genes, was provided by Tzyy-Choou Wu (Johns Hopkins University) and cultured in cRPMI in the presence of 100 μg/mL G418 (Geneticin, GIBCO) to select for transfectants at 37°C and 5% CO_2_. Cells were harvested by trypsinization (GIBCO) and washed twice with PBS and suspended at a concentration of 1 × 10^6^ cells/mL in PBS. B16-F10 or TC-1 cells (1 × 10^5^ each) were injected s.c. into the flanks of mice. Health, weight, and tumor size were tracked at least twice per week until the endpoint of the study. Tumor size was measured by long and short length, and tumor volume was obtained by the flowing formula: volume = length × width^2^ × π/6.

### In vivo BMDC treatments.

Four days after B16-F10 tumor cell inoculation, mice received the first dose of 2 × 10^6^ hgp100_25–33_ peptide–pulsed mBMDC vaccine intradermally (i.d.) and the vaccination was repeated with 3 more doses at 5-day intervals. Eight days after TC-1 tumor cell inoculation, mice received the first dose of 2 × 10^6^ E7_43–77_ peptide–pulsed mBMDC vaccine i.d. and the vaccination was repeated with 4 or 5 doses at 5-day intervals. mBMDCs (2 × 10^6^) or a mixture of 2 × 10^6^ E7_43–77_-pulsed WT mBMDCs and 2 × 10^6^ cells of either of the WT or bm12 mBMDCs were injected i.d. in TC-1 tumor–bearing mice for each of 4 doses starting 8 days after TC-1 cell inoculation at 5-day intervals.

### In vivo depletion.

To deplete CD4^+^ or CD8^+^ T cells, WT mice were injected intraperitoneally (i.p.) with 200 μg of anti-CD4 antibody (clone GK1.5, Bio X Cell), anti-CD8 antibody (clone 53-6.7, Bio X Cell), or IgG2a isotype control (Bio X Cell) as a first dose, and 100 μg of each antibody was injected every 2–3 days from a second dose continuing until the end of the study. To deplete Foxp3^+^ Tregs, Foxp3-GFP^DTR^ mice were injected i.p. with 10 μg/kg of DT every 2–3 days continuing until the end of the study. As early depletion of CD4^+^ T cells, CD8^+^ T cells, or Foxp3^+^ Tregs, injection of either antibody or DT was started on day 6 after TC-1 inoculation (2 days before the first dose of BMDC vaccination). For late depletion of either CD4^+^ T cells or Foxp3^+^ Tregs, injection of either antibody or DT was started on day 15 after TC-1 inoculation (2 days after the second dose of BMDC vaccination).

### Isolation of CD4^+^ and CD8^+^ T cells.

To obtain splenocytes, spleens were minced over a 70 μm filter, spun down, and red blood cells were removed using ACK lysing buffer (Lonza). Single-cell suspension was passed through a 40 μm filter. Pmel-1 CD8^+^ T cells for coculturing with BMDCs were isolated from female Pmel-1 mouse splenocytes using a CD8a^+^ T cell Isolation kit (Miltenyi Biotec) according to the manufacturer’s directions. CD4^+^ and CD8^+^ T cells for coculturing with BMDCs were isolated from spleens of female TC-1 tumor–bearing mice 8–12 days after TC-1 tumor cell inoculation using a CD4^+^ T cell Isolation kit (Miltenyi Biotec) or CD8a^+^ T cell Isolation kit (Miltenyi Biotec) according to the manufacturer’s directions.

### MHC class II blocking.

The WT or bm12 BMDCs were blocked with 20 μg/mL anti–MHC class II antibody (clone M5/114.15.2, Invitrogen) for 1 hour according to the manufacturer’s directions and washed with PBS.

### ELISA.

Supernatants from 24-hour cocultures of Pmel-1 CD8^+^ T cells and 72-hour cocultures of TC-1 tumor–bearing mouse splenic CD4^+^ T cells or CD8^+^ T cells were collected and used in ELISA to measure secreted IFN-γ using the DuoSet kit (R&D Systems) according to the manufacturer’s instructions. The samples were analyzed in triplicate. To collect plasma of DT-administered Foxp3-GFP^DTR^ mice, blood was drawn in EDTA-coated tubes, the cells were spun down, and the supernatant was collected. Plasma was used in ELISA to measure anti-DT IgG using the Mouse Anti-Diphtheria Toxin/Toxoid IgG ELISA kit (Genemed Synthesis, Inc.) according to the manufacturer’s instructions. The samples were analyzed in triplicate.

### Flow cytometry.

To obtain single-cell suspensions from spleens and blood for flow cytometric analysis, spleens were processed against a 70 μm filter, spun down, then red blood cells were removed using ACK lysing buffer (Lonza) and passed through a 40 μm filter and washed with PBS. To obtain tumor-infiltrating cells, tumors were harvested, fat and necrotic tissue was removed, and the tumors were dissociated using the gentleMACS tissue dissociator with the Tumor Dissociation Kit (Miltenyi Biotec) according to the manufacturer’s directions. A single-cell suspension was prepared by Ficoll-Paque density gradient centrifugation and washed with PBS. To detect IFN-γ–expressing CD4^+^ or CD8^+^ T cells in cocultures with BMDCs, 1 μL/mL brefeldin A (BD Biosciences) was added 6 hours before harvesting the cells. BMDCs or single-cell suspensions from spleens, blood, and tumors for flow cytometry were incubated with anti-CD16/anti-CD32 (Fc block, BD Biosciences) on ice and Live/Dead fixable blue dead cell stain (Invitrogen) for 20 minutes at room temperature. Cells were then labeled for surface marker antibodies on ice for 30 minutes and fixed/permeabilized using a BD Cytofix/Cytoperm solution kit (BD Biosciences) for detection of intracellular proteins according to the manufacturer’s instructions. Cells were then washed and labeled with intracellular antibodies. All antibodies used for staining cells in this study are listed in Supplemental Table 1. The stained samples were acquired on a BD FACSymphony A5, and the flow cytometry data were analyzed using FlowJo (version 10) (FlowJo LLC).

### Statistics.

Data are expressed as the mean ± SEM for each group. Comparisons involving more than 2 groups were performed using a 1- or 2-way analysis of variance (ANOVA) test with post hoc Tukey’s multiple-comparison correction, and correlation between 2 subjects was performed using linear regression analysis and survival differences of tumor-bearing mice were assessed using Kaplan-Meier curves and analyzed by log-rank tests using GraphPad Prism (version 10). *P* values of less than 0.05 were considered statistically significant.

### Study approval.

All animal procedures reported in this study that were performed by the NCI Center for Cancer Research–affiliated staff were approved by the NCI Animal Care and Use Committee (ACUC) and in accordance with federal regulatory requirements and standards (reference number: METB-033 and MOB-032). All parts of the intramural NIH ACUC program are accredited by AAALAC International.

### Data availability.

The authors confirm that the data supporting the findings of this study are available within the article and in its supplemental material. Values underlying the data presented in each graph and as means are provided in the Supplemental Supporting Data Values file. Data sets analyzed during the current study are available from the corresponding author upon request.

## Author contributions

NS, WB, PBO, HMM, MALL, WVW, and JAB designed the study. NS and WB collected the data. NS, WB, and JAB analyzed the data. NS, WB, PBO, HMM, MALL, WVW, and JAB interpreted the data and discussed the conclusions. NS, WB, and JAB wrote the manuscript, which all authors critiqued and edited.

## Supplementary Material

Supplemental data

Supporting data values

## Figures and Tables

**Table 1:**
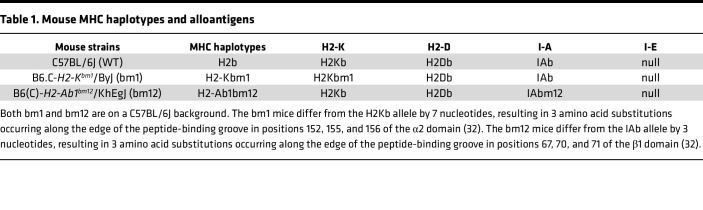
Mouse MHC haplotypes and alloantigens

## Version 1 08/22/2025

Electronic publication
