# Supplementary material for: Enhancing DC cancer vaccine by allogeneic MHC class II expression and Treg depletion
Source: JCI Insight. 2025 Jul 15;10(16):e189024. doi: 10.1172/jci.insight.189024 (PMC12406729; doi:10.1172/jci.insight.189024)
Supplement: Supplemental data [file jciinsight-10-189024-s030.pdf]

Supplementary Figure S1

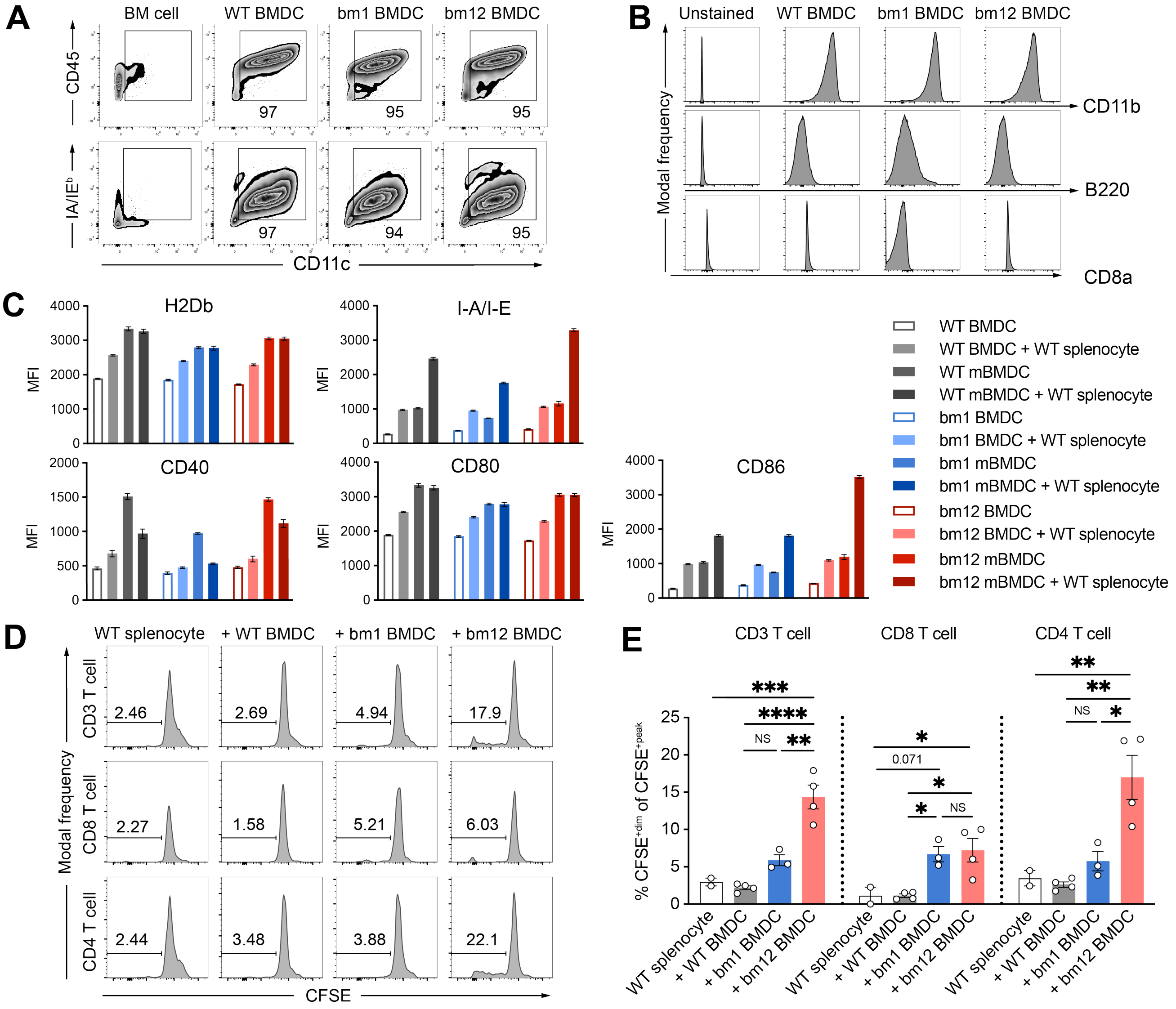

Supplementary Figure S1. Syngeneic, MHC class I semi-allogeneic and MHC class II semi-allogeneic BMDCs express MHC I/ II and costimulatory molecules similarly, but MHC class II semi-allogeneic BMDCs enhance WT CD4$^+$ T cell proliferation. (A, B) Representative examples of (A) CD45 and MHC II$^+$ expression on CD11c$^+$ cells and (B) expression of CD11b, B220 and CD8a on syngeneic WT, MHC class I semi-allogeneic bm1, and MHC class II semi-allogeneic bm12 BMDCs. WT, bm1, and bm12 BMDCs were generated by culturing BM cells with 20 ng/ml GM-CSF for 10 days and analyzed by flow cytometry. WT BM cells were used for gating of CD11c and IA/IE. Numbers in the zebra plots indicate the percentage of gated cells. (C) Median fluorescence intensity (MFI) of indicated cell surface molecules on the BMDCs in various MLR settings. $1x10^5$ cells of BMDCs or LPS-matured BMDCs (mBMDCs) were cultured alone or cocultured with $4x10^5$ WT splenocytes in 200 µl/well in 96-well round bottom plates for 18 hours and analyzed by flow cytometry. (D, E) WT T cell proliferation induced by syngeneic mixed lymphocyte reaction (MLR) or semi-allogeneic MLR. $4x10^5$ WT splenocytes stained with 0.1mM CFSE from naïve mice were cultured alone or cocultured with $1x10^5$ cells of the WT, bm1 or bm12 BMDCs in 200 µl/well in 96-well round bottom plates for 5 days and analyzed by flow cytometry. (D) Representative examples of proliferation rate of CD3$^+$, CD8$^+$, or CD4$^+$ T cells gating on the CFSE dim region. Numbers in the histogram indicate the percentage of proliferating cells. (E) Individual T cell proliferation values. Results were assessed in each comparison over CD3$^+$, CD8$^+$, or CD4$^+$ T cell respectively using the one-way ANOVA test with post hoc Tukey's multiple comparison corrections. n=4 (C), 2–4 (D, E) per group. Data are represented as the mean ± SEM and represent at least 3 independent experiments. *P < 0.05; **P < 0.01; ***P < 0.001; ****P < 0.0001.

Supplementary Figure S2

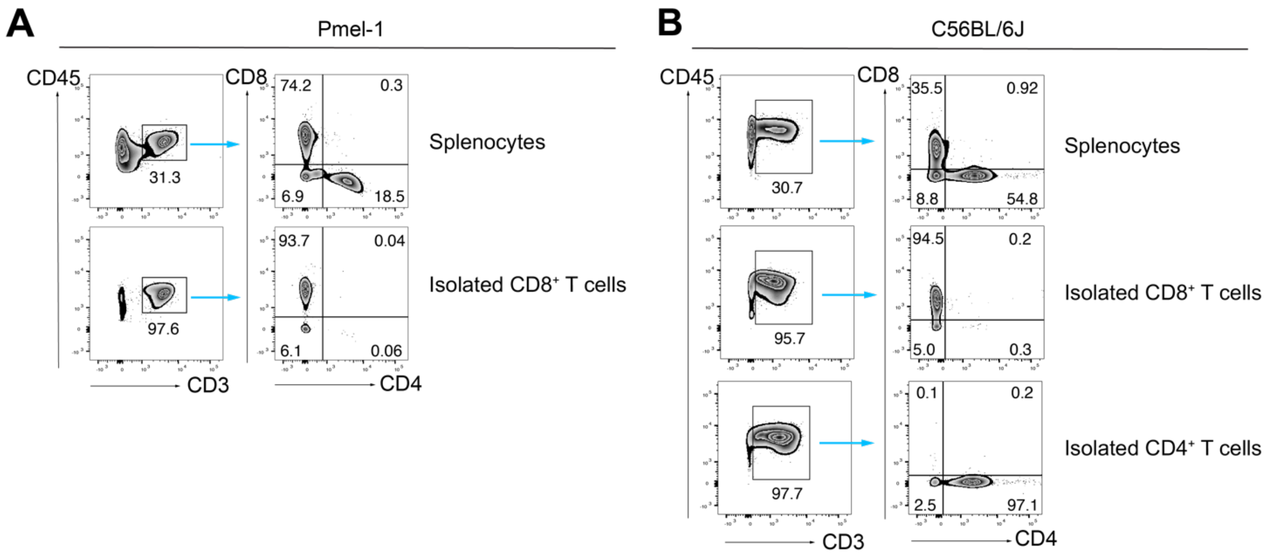

Supplementary Figure S2. Purity of isolated CD8$^+$ T cells and CD4$^+$ T cells used in vitro studies. Comparison of splenocytes and (A) isolated CD8$^+$ T cells from Pmel-1 mice for in vitro studies in Figure 1B and (B) isolated CD8$^+$ T cells and CD4$^+$ T cells from C56BL/6J mice for in vitro studies in Figures 3 and 4. (A) Female Pmel-1 mice aged 8–12 weeks old were used for preparing splenocytes. (B) Splenocytes from female C56BL/6J WT mice aged 8–10 were harvested 8–12 days after 1x10^5 TC-1 inoculation for preparing splenocytes. CD4$^+$ or CD8$^+$ T cells from the mouse splenocytes were isolated using CD4$^+$ T cell Isolation kit (Miltenyi Biotec) or CD8a$^+$ T cell Isolation kit (Miltenyi Biotec) according to manufacturer's directions. Purity of isolated T cells was analyzed by flow cytometry in at least 3 independent experiments. Numbers in the zebra plots indicate the percentage of gated cells.

Supplementary Figure S3

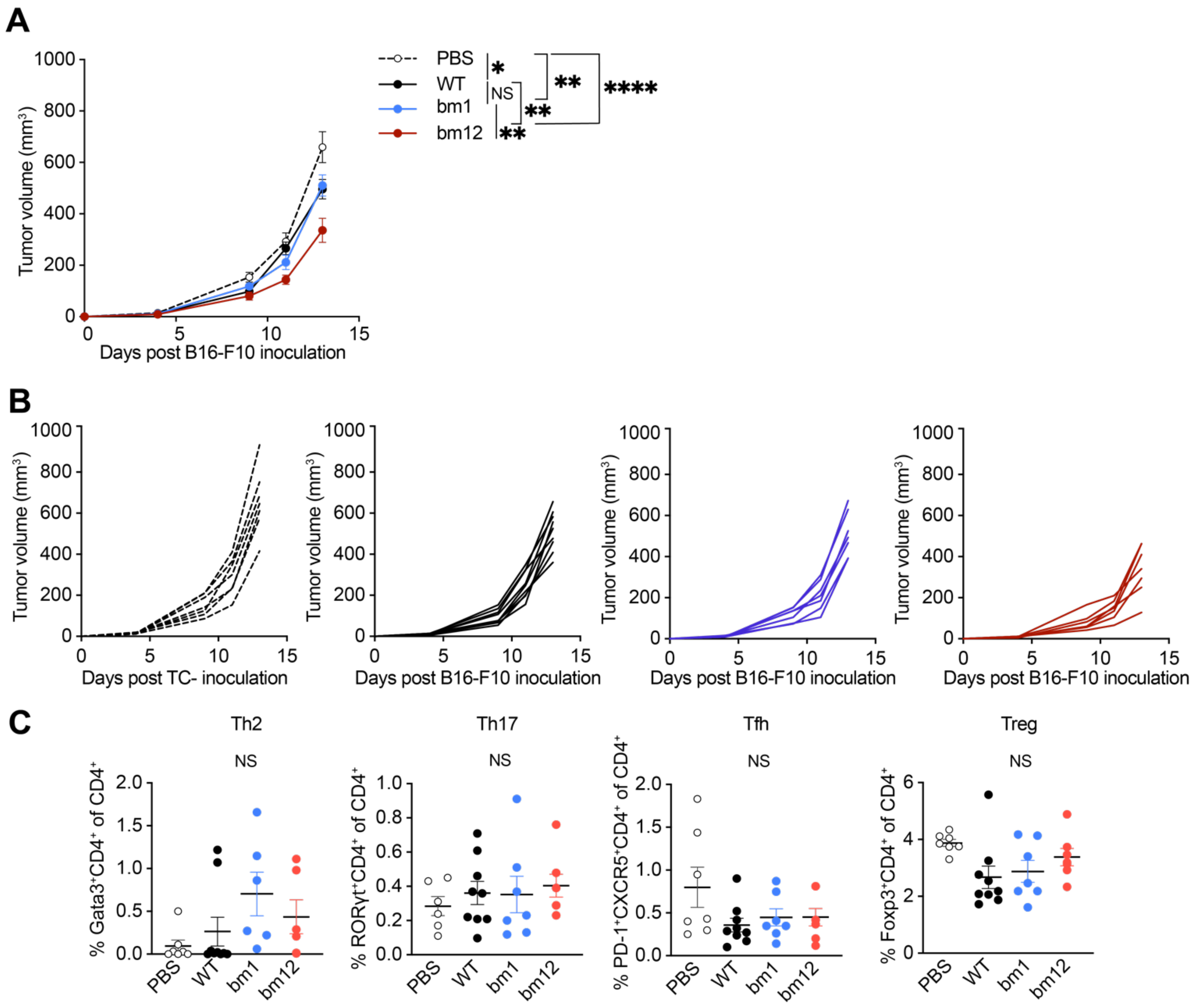

Supplementary Figure S3. (A, B) The hgp100$_{25-33}$ peptide-pulsed syngeneic WT, MHC class I semi-allogeneic bm1, or MHC class II semi-allogeneic bm12 mature BMDC vaccine efficacy in B16-F10 until sacrificing mice on day 14 after tumor inoculation and (C) CD4 subset changes in tumors by flow cytometry. (A, B) WT mice were injected s.c. with $1x10^5$ B16-F10 tumor cells, and mice were injected intradermally (i.d.) with either of the hgp100$_{25-33}$-pulsed WT, bm1, or bm12 mBMDC vaccines three times starting 4 days after tumor inoculation at 5-day intervals until sacrificing on day 14 after tumor inoculation. (A) Average tumor growth and (B) individual tumor growth of indicated groups. (C) Individual mouse values of Th2, Th17, Tfh cells and Tregs in tumors. The mice noted in A and B were sacrificed 14 days after B16-F10 tumor inoculation, and tumors were collected and subjected to flow cytometric analysis of $CD4^+$ T cell subsets in tumors. Data were represented as the mean ± SEM and represent 3 independent experiments, n=6–9 per group. Statistical analysis was performed using two-way ANOVA test with post hoc Tukey's multiple comparison correction.

Supplementary Figure S4

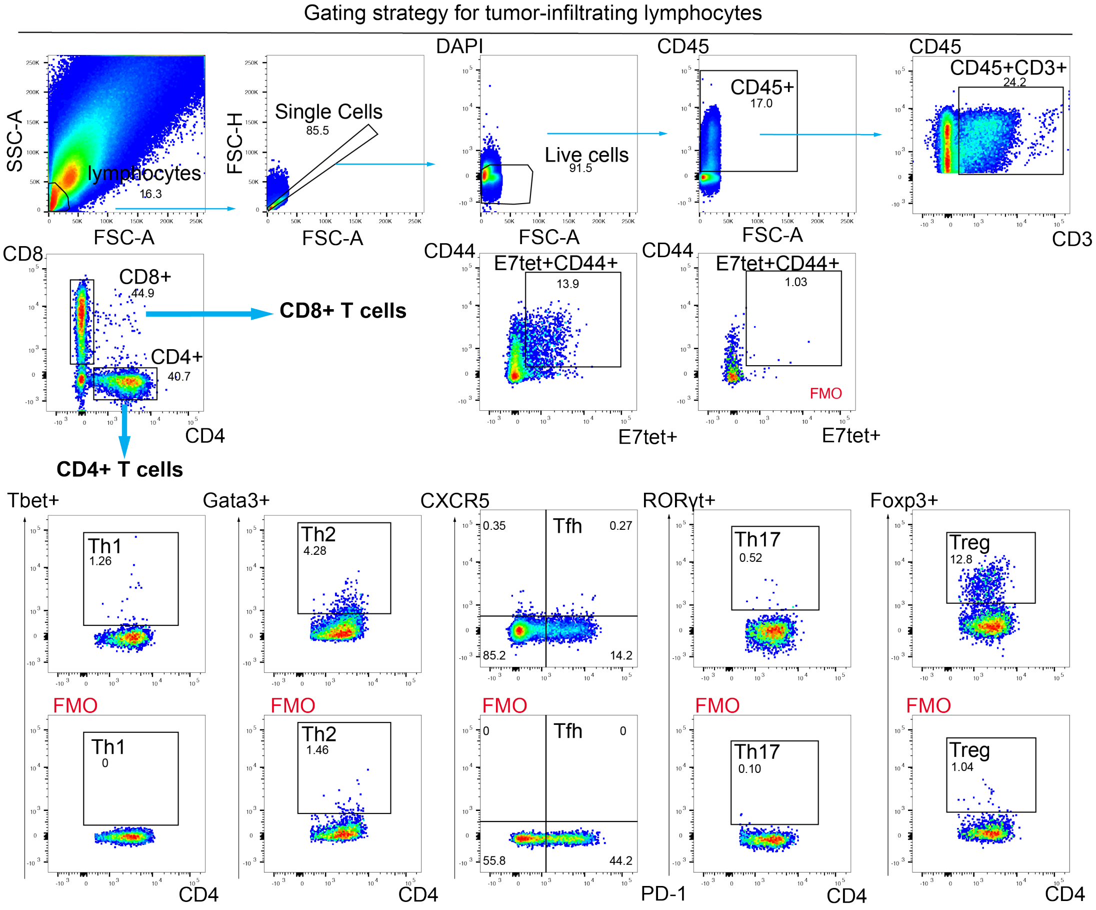

Supplementary Figure S4. Gating strategy for $CD8^+$ T cells and $CD4^+$ T cells in tumors. WT mice were injected s.c. with $1x10^5$ TC-1 tumor cells on day 0, and mice were injected i.d. with either of the $E7_{43-77}$-pulsed syngeneic WT, MHC class I semi-allogeneic bm1, or MHC class II semi-allogeneic bm12 mBMDC vaccines four times starting on 8-day after TC-1 inoculation with 5-day intervals. Mice were sacrificed 22 days after TC-1 inoculation, and tumors were collected and subjected to flow cytometric analysis of infiltrating $CD8^+$ and $E7/H2D^b$ tetramer$^+$ cells $CD4^+$ T cell subsets in tumors.

Supplementary Figure S5

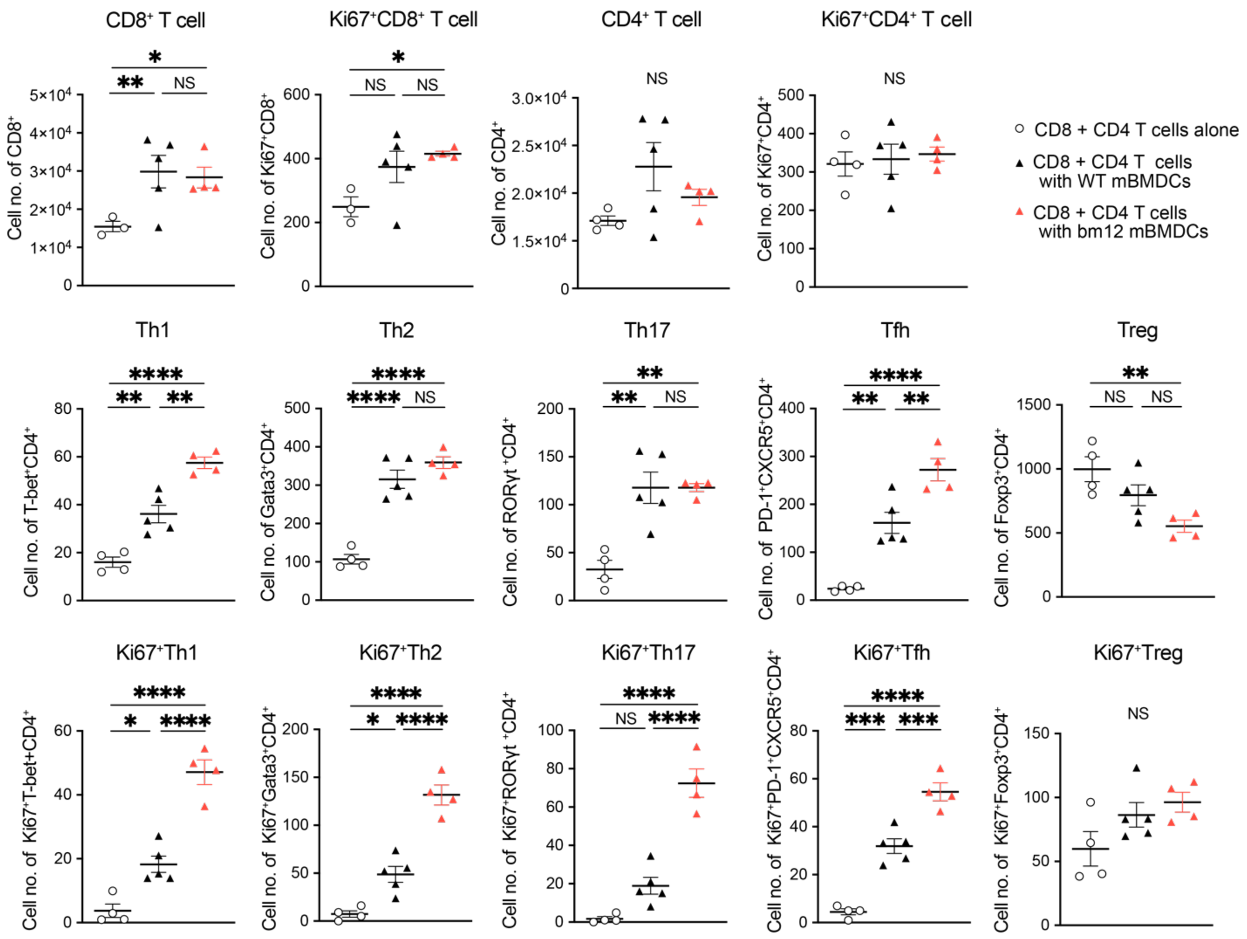

Supplementary Figure S5. $E7_{43\text{-}77}$-pulsed MHC class II semi-allogeneic mature BMDCs expand Th1, Th2, Tfh, and Th17 cells but reduce the proportion of Treg cells. In vitro $CD8^+$ or $CD4^+$ T cell subset changes after coculture of mixtures of $CD8^+$ T cell and $CD4^+$ T cell from TC-1 tumor-bearing mice stimulated with either of the syngeneic WT or MHC class II semi-allogeneic bm12 LPS-matured BMDCs (mBMDCs). TC-1 tumor-bearing mice were sacrificed 8–12 days after inoculation of $1x10^5$ TC-1 cells and mixtures of $2x10^5$ isolated $CD8^+$ T cells and $2x10^5$ isolated $CD4^+$ T cells were cocultured with $1x10^5$ of the indicated mBMDCs in 200 ul/well of 96-well round bottom plates for 48 hours and subjected to flow cytometry analysis. Data are represented as the mean ± SEM and represent 2 independent experiments, n=4–5 per group. Statistical analysis was performed using one-way ANOVA test with after hoc Tukey's multiple comparison corrections. *$p<0.05$; **$p<0.01$; ***$p<0.001$; ****$p<0.0001$.

Supplementary Figure S6

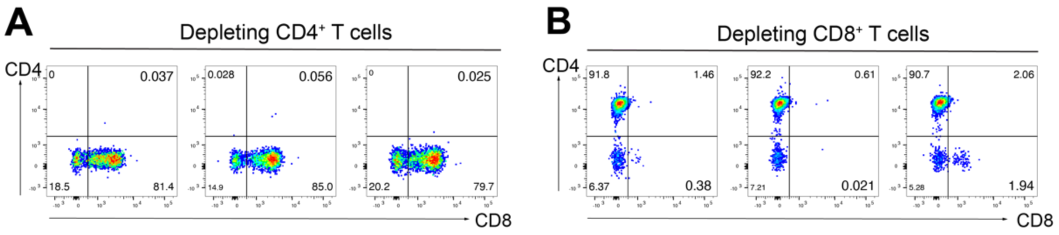

Supplementary Figure S6. Confirming CD4$^+$ and CD8$^+$ T cell depletion on day 7 after TC-1 inoculation. To deplete CD4$^+$ T cells or CD8$^+$ T cells, WT mice were injected intraperitoneally initially with 200 ug of anti-CD4 antibody (clone: GK1.5, BioXcell), anti-CD8 antibody (clone: 53-6.7, BioXcell), or IgG2a isotype control (BioXcell), and 100 ug of each antibody every 2–3 days subsequently continuing until the end of the study. For early depletion of either CD4$^+$ T cells or CD8$^+$ T cells, injection of either antibody was started on day 6 after TC-1 inoculation (two days before the first dose of BMDC vaccinations). For late depletion of either CD4$^+$ T cells or CD8$^+$ T cells, injection of either antibody started on day 15 after TC-1 inoculation (two days after the second dose of BMDC vaccinations) as Late depletion. (A, B) Individual examples of splenocytes in the blood of mice from depletion of either (A) CD4$^+$ T cells, n=3 or (B) CD8$^+$ T cells, n=3 on day 7 post-TC-1 inoculation. Depletion of CD4$^+$ T cells and CD8$^+$ T cells were confirmed by flow cytometry on day 7- and day 15- after TC-1 inoculation from the mice for the early depletion.

Supplementary Figure S7

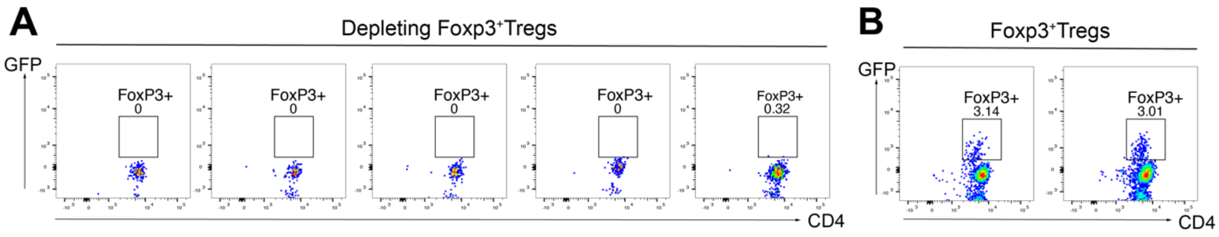

Supplementary Figure S7. Confirming Foxp3^{+} Treg depletion. To deplete Foxp3^{+} Tregs, Foxp3-GFP^{DTR} mice were injected intraperitoneally with 10 µg/ kg of human diphtheria toxin (DT) every 2–3 days continuing until the end of the study. As early depletion of Foxp3^{+} Tregs, injection of either antibody or DT was started on day 6 after TC-1 inoculation (two days before the first dose of BMDC vaccination). As late depletion of Foxp3^{+}Tregs, injection of either antibody or DT was started on day 15 after TC-1 inoculation on day 15 after TC-1 inoculation (two days after the second dose of BMDC vaccinations) as Late depletion. (A, B) Individual examples of splenocytes in the blood of mice from (A) depletion of Foxp3^{+}Tregs, n=5 or (B) no-depletion of Foxp3^{+}Treg, n=2. (A) Depletion of Foxp3^{+} Tregs were confirmed by flow cytometry on day 9 after TC-1 inoculation from the mice grouped for the early depletion compared to (B) the mice grouped for PBS.

Supplementary Figure S8

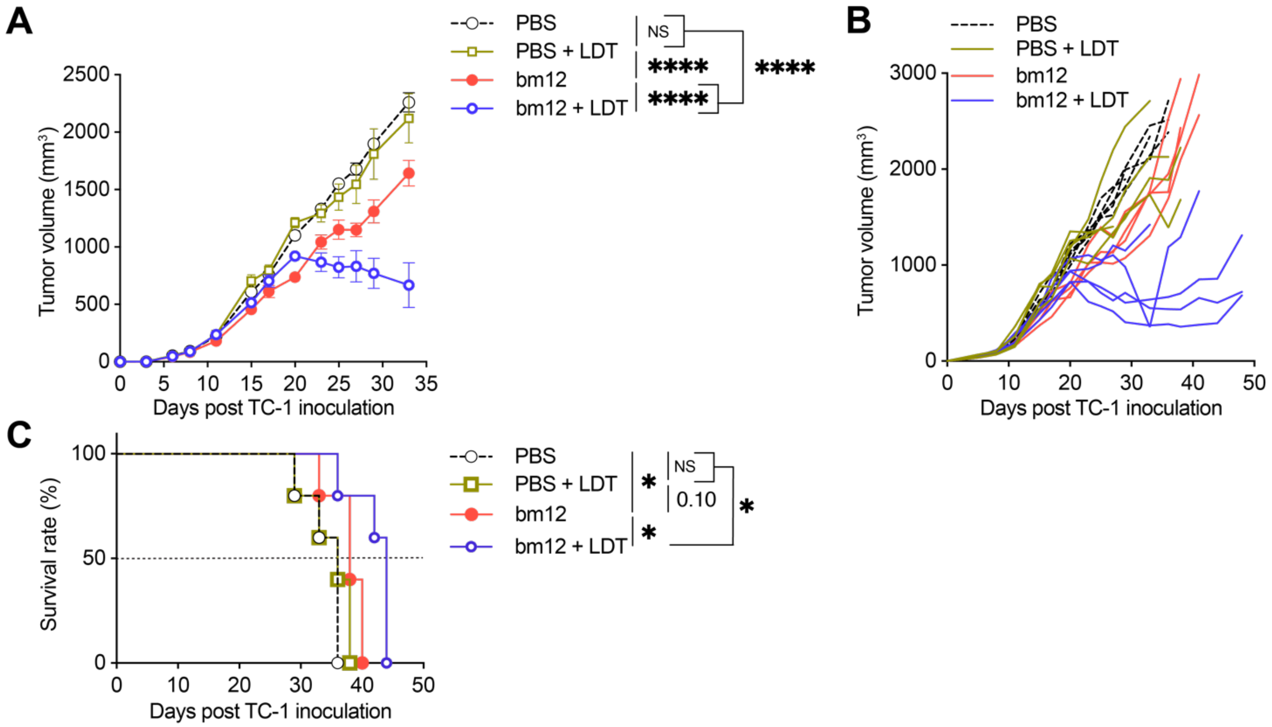

Supplementary Figure S8. Late Treg depletion alone has no effect on delaying TC-1 tumor growth. (A–C) Foxp3-GFP^{DTR} mice were injected s.c. with 1x10^5 TC-1 tumor cells, and mice were injected intradermally (i.d.) with E7$_{43\text{-}77}$-pulsed MHC class II semi-allogeneic bm12 LPS-matured BMDC (mBMDC) vaccines (designated bm12 in the graphs) five times starting 8 days after tumor inoculation at 5-day intervals. Tregs were depleted by intraperitoneal injection (i.p.) of diphtheria toxin (DT) that started two days after the second dose of the BMDC vaccinations as late depletion (LDT) and continued every 2–3 days until end of the study. (A) Average tumor growth of indicated groups and (B) individual tumor growth of each tumor-bearing mouse. (C) Survival rate of indicated groups. The median value of each group; PBS, 36 days; PBS + LDT, 36 days; bm12, 38 days; bm12 + LDT, 44 days. Data are represented as the mean ± SEM and represent 2 independent experiments, n=4–6 per group. Statistical analysis was performed using two-way ANOVA test with post hoc Tukey's multiple comparison corrections over the entirety of the study (A) and Kaplan-Meier curves and analyzed by log-rank (C). *p<0.05; ****p<0.0001.

Supplementary Figure S9

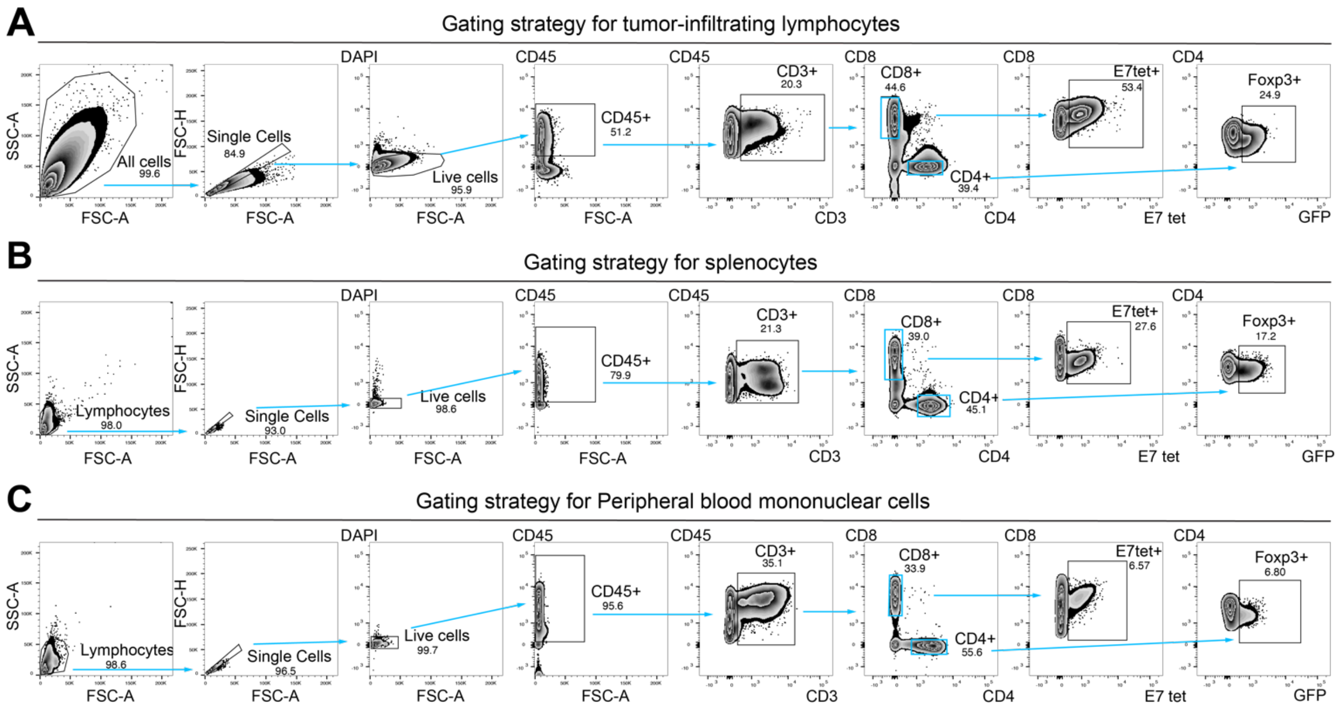

Supplementary Figure S9. Gating strategy for E7/H-2D^b tetramer$^+$ CD8$^+$ T cells and Foxp3$^+$ CD4$^+$ T cells in tumors, spleens and blood. Foxp3-GFP^{DTR} mice were injected s.c. with 1x10^5 TC-1 tumor cells on day 0, and mice were injected intradermally (i.d.) with E7$_{43-77}$-pulsed MHC class II semi-allogeneic bm12 LPS-matured BMDC (mBMDC) vaccines five times starting 8 days after TC-1 inoculation at 5-day intervals. Tregs were depleted by intraperitoneal injection of human diphtheria toxin (DT) that started two days before the TC-1 inoculation for early depletion or two days after the second dose of the BMDC vaccinations for late depletion and continued every 2–3 days until end of the study. Gating strategy for E7/H2Db tetramer$^+$CD8$^+$ T cells and Foxp3$^+$ CD4$^+$ T cells in (A) tumors, (B) spleens and (C) blood.

Supplementary Figure S10

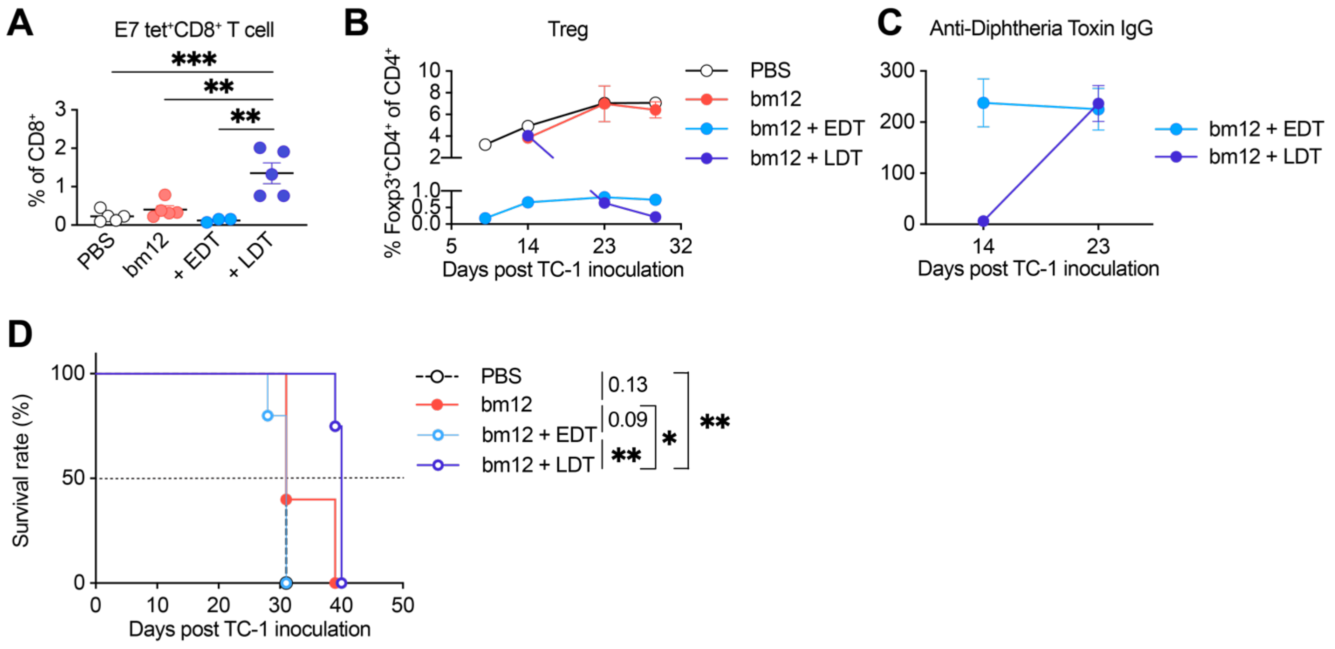

Supplementary Figure S10. (A) Average E7/H-2D^{b} tetramer^{+} CD8^{+} T cell 29 days after TC-1 inoculation. (B) Average Foxp3^{+} Treg change in blood by flow cytometry at different time points. (C) Anti-DT IgG level in plasma by ELISA 14 and 23 days after TC-1 inoculation. (D) Survival rate. Foxp3-GFP^{DTR} mice were injected s.c. with 1x10^{5} TC-1 tumor cells on day 0, and mice were injected i.d. with E7$_{43\text{-}77}$-pulsed MHC class II semi-allogeneic bm12 mBMDC vaccines (designated bm12 in the graphs) five times starting 8 days after tumor inoculation at 5-day intervals. Tregs were depleted by intraperitoneal injection of diphtheria toxin (DT) that started two days before the TC-1 inoculation for early depletion (EDT) or two days after the second dose of the BMDC vaccinations for late depletion (LDT) and continued every 2–3 days until end of the study. (A, B) Mouse blood was subjected to flow cytometric analysis of (A) CD8^{+} and E7/H2Db tetramer^{+} cells and (B) Foxp3^{+} CD4 T cells. (D) The median value of each group; PBS, 31 days; bm12, 31 days; bm12 + EDT, 31 days; bm12 + LDT, 40 days. Data are represented as the mean ± SEM (A, B), n=3–5 per group (A, B, D) and triplicate of n=4–6 (C).

Supplementary Table S1: Flow cytometry antibodies

Data location	Antigen	Fluorophore	Clone	Catalog #	Company
Figure 2–6, Supplementary Figure S3–S8	CD45	APC-R700	30-F11	560510	BD
	CD3	PE	145-2C11	100308	BioLegend
	CD4	BUV496	GK1.5	612952	BD
	CD8a	BB630	53-6.7	624294	BD
	CD44	APC-Cy7	IM7	560568	BD
	E7 tetramer	BV421	Sequence: RAHYNIVTF		NIH Tetramer core
	hgp100 tetramer	BV421	Sequence: KVPRNQDWL		NIH Tetramer core
	PD-1	BV605	29F.1A12	135220	BioLegend
	Ki67	PerCp-Cy5.5	B56	561284	BD
	T-bet	BV711	O4-46	563320	BD
	Gata3	APC	16E10A23	653806	BioLegend
	CXCR5 (CD185)	BV786	L138D7	145523	BioLegend
	RORγt	BV480	Q31-378	567176	BD
	Foxp3	FITC	FJK-16s	11-5773-82	Invitrogen
	IFNγ	APC	XMG1.2	554413	BD
Supplementary Figure S1, S2	CD45	APC-R700	30-F11	560510	BD
	$H2D^b$	FITC	KH95	562000	BD
	IA/IE^b	BV421	M5/114.15.2	562564	BD
	CD40	Alexa Fluor 647	HM40-3	102912	BD
	CD80	PerCP-Cy5.5	16-10A1	104722	BioLegend
	CD86	PE	GL1	553692	BD
	CD11c	PE-Cy7	HL3	558079	BD
	CD11b	APC-Cy7	M1/70	A15390	Invitrogen
	CD8a	BV650	RPA-T8	563822	BD
	B220	BV786	RA3-6B2	563894	BD
	CD3	PE	145-2C11	100308	BioLegend
	CD4	APC-Cy7	GK1.5	100414	BD
	CD8a	PerCP-Cy5.5	53-6.7	100734	BioLegend
